# Spermatid Cyst Polarization in *Drosophila* Depends upon *apkc* and the CPEB Family Translational Regulator *orb2*

**DOI:** 10.1371/journal.pgen.1004380

**Published:** 2014-05-15

**Authors:** Shuwa Xu, Sanjay Tyagi, Paul Schedl

**Affiliations:** 1 Department of Molecular Biology, Princeton University, Princeton, New Jersey, United States of America; 2 Public Health Research Institute, New Jersey Medical School, Rutgers University, Newark, New Jersey, United States of America; 3 Institute of Gene Biology, RAS, Moscow, Russia; Weizmann Institute of Science, Israel

## Abstract

Mature *Drosophila* sperm are highly polarized cells—on one side is a nearly 2 mm long flagellar tail that comprises most of the cell, while on the other is the sperm head, which carries the gamete's genetic information. The polarization of the sperm cells commences after meiosis is complete and the 64-cell spermatid cyst begins the process of differentiation. The spermatid nuclei cluster to one side of the cyst, while the flagellar axonemes grows from the other. The elongating spermatid bundles are also polarized with respect to the main axis of the testis; the sperm heads are always oriented basally, while the growing tails extend apically. This orientation within the testes is important for transferring the mature sperm into the seminal vesicles. We show here that orienting cyst polarization with respect to the main axis of the testis depends upon atypical Protein Kinase C (aPKC), a factor implicated in polarity decisions in many different biological contexts. When *apkc* activity is compromised in the male germline, the direction of cyst polarization within this organ is randomized. Significantly, the mechanisms used to spatially restrict *apkc* activity to the apical side of the spermatid cyst are different from the canonical cross-regulatory interactions between this kinase and other cell polarity proteins that normally orchestrate polarization. We show that the asymmetric accumulation of aPKC protein in the cyst depends on an mRNA localization pathway that is regulated by the *Drosophila* CPEB protein Orb2. *orb2* is required to properly localize and activate the translation of *apkc* mRNAs in polarizing spermatid cysts. We also show that *orb2* functions not only in orienting cyst polarization with respect to the apical-basal axis of the testis, but also in the process of polarization itself. One of the *orb2* targets in this process is its own mRNA. Moreover, the proper execution of this *orb2* autoregulatory pathway depends upon *apkc*.

## Author Summary

After completion of meiosis, the 64 cells in the spermatid cyst begin differentiating into sperm. Sperm are highly polarized cells and a critical step in their differentiation is spermatid cyst polarization. Spermatids are also polarized within the testis, with the heads of the elongating spermatids located basally, while the tails extend apically. We show that aPKC accumulates preferentially on the apical side of the cyst during polarization and is required to correctly orient cyst polarization with respect to the apical-basal axis of the testis. Unexpectedly, aPKC activity is spatially restricted by a mechanism that depends upon the CPEB family translational regulator *orb2*. *orb2* is required to asymmetrically localize and activate the translation of *apkc* mRNAs during spermatid differentiation. In addition to correctly orienting the direction of cyst polarization, *orb2* is required for the process of polarization itself. One of the *orb2* regulatory targets in this process is its own mRNA, and this autoregulatory activity depends, in turn, upon *apkc*.

## Introduction

Polarity plays a central role in a diverse array of biological contexts in organisms ranging from single cell bacteria to complex multicellular eukaryotes. In multicellular eukaryotes, the steps involved in establishing, maintaining, and transmitting polarity are typically controlled by an interacting set of evolutionarily conserved atypical protein kinase C-partitioning defective proteins (aPKC-PAR proteins). The classic model for polarity determination by the aPKC-PAR machinery is the establishment of the anterior-posterior axis in the *C. elegans* embryo [1]. Prior to fertilization, anterior determinants, the worm aPKC ortholog PKC-3, PAR-3 and PAR-6, are distributed in a complex around the entire cortex of the egg [2]–[4], while the posterior factors, PAR-1 and PAR-2, are cytoplasmic. PAR-2 is kept off the cortex by PKC-3 dependent phosphorylation, and a similar mechanism may apply to PAR-1 [5], [6]. Sperm entry induces a cytoplasmic flux that relocalizes the PKC-3/PAR-3/PAR-6 complex in the posterior to the anterior cortex. Following the removal of PKC-3 activity from the posterior, PAR-1 and PAR-2 are able to associate with the cortex. Cortical PAR-2 in turn prevents re-association of anterior determinants with the posterior cortex (for review: [7]). This generates a polarized cell in which the PKC-3/PAR-3/PAR-6 complex is distributed along the anterior cortex, while PAR-1/PAR-2 are localized on the posterior cortex. This process also serves to orient the mitotic spindle: the first cell division in the embryo is parallel to the anterior-posterior axes and as a consequence the two daughter cells receive different sets of embryonic determinants [1]. The aPKC-PAR machinery defines polarity in many other contexts besides the establishment of the anterior-posterior axis of the *C.elegans* embryo. Moreover, as in *C.elegans*, antagonistic interactions between aPKC-PAR family proteins are critical for establishing and propagate cell asymmetry in many systems (for reviews: [8]–[11].

**Figure 1:**
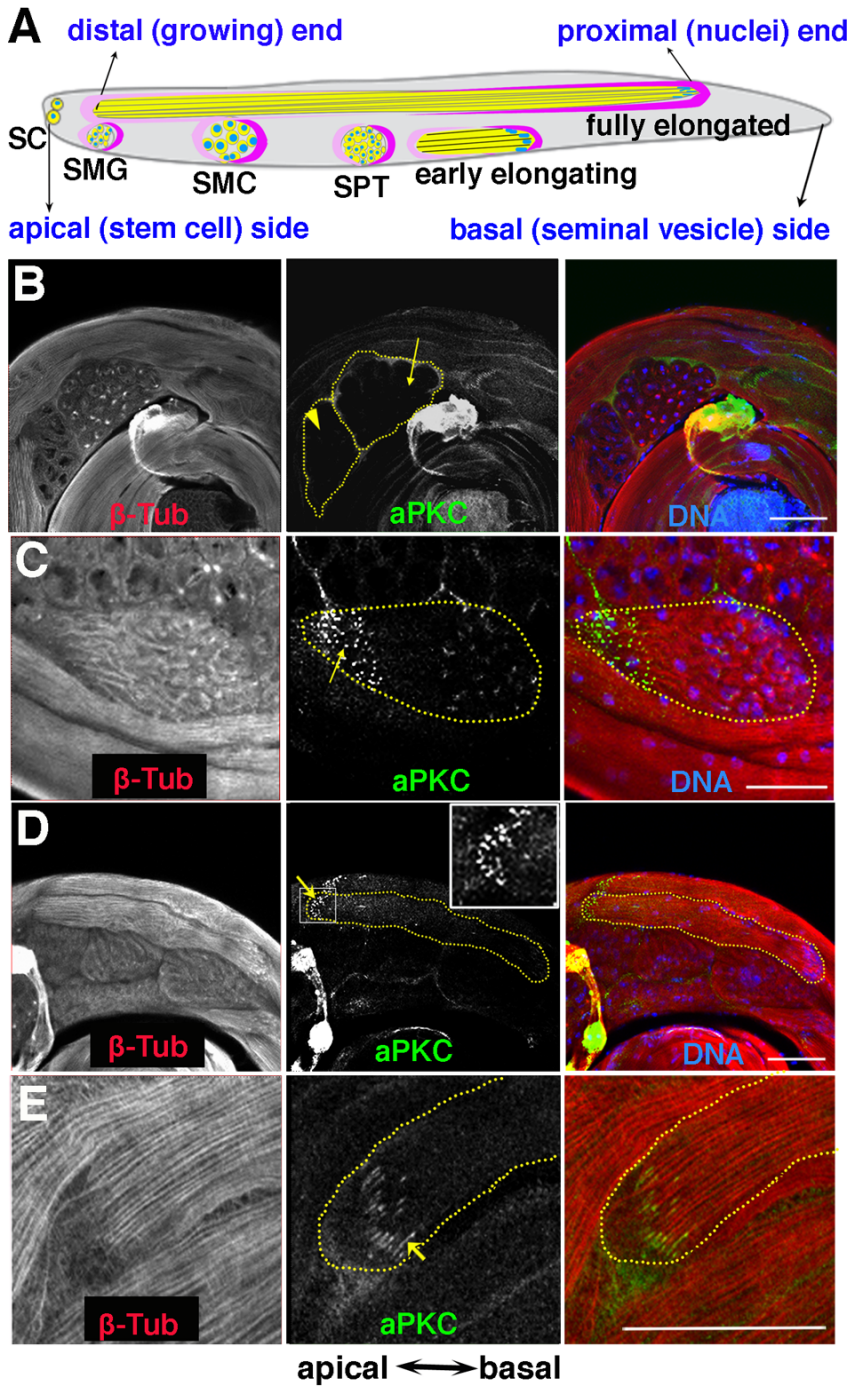
aPKC expression in spermatid cysts. A) Stages of spermatogenesis. Germline cysts are in yellow, while the somatic cyst cells are labeled in dark pink (posterior or head cyst cell) and light pink (anterior or tail cyst cell) respectively. SC, stem cell; SMG, spermatogonia; SMC, spermatocyte; SPT, spermatid. B–E) aPKC expression during spermatogenesis. B) Localized aPKC is not observed in pre-meiotic (arrowhead) or meiotic (arrow) cysts. C) When the spermatid cyst polarizes, aPKC puncta (green) are clustered on the distal side of the polarizing cyst while the spermatid nuclei localize on the proximal side of the cyst. D, E) aPKC stripes at the tip of the growing flagellar axoneme (arrow). Insert in D is a zoom-in view of the marked window. All images are orientated with the apical end of the testes to the left and the basal to the right. Scale bar: 50 µm.

In most of the well-studied model systems, the aPKC-PAR machinery is deployed to generate undifferentiated cells that are then able to assume different identities. However, polarity can also be a distinguishing feature of terminally differentiated cells. One example of a highly polarized fully differentiated cell is the mature *D. melanogaster* sperm. At one end of the mature sperm cell is the sperm head, which contains the highly condensed haploid genome encased in a multilayer membrane. The rest of the cell is the nearly 2 mm long flagellar axoneme tail, which is connected to the head by a centrosome-derived structure called the basal body. The formation of this polarized cell commences after meiosis is completed and the 64 interconnected spermatids begin the process of differentiation (Fig. 1A). Each haploid nucleus has a single basal body with a short axoneme surrounded by a membrane cap. In the first steps the basal body inserts into the nuclear envelope, where it functions to nucleate the assembly of the flagellar axoneme. The 64-cell cyst re-organizes so that the spermatid nuclei cluster together on the proximal or basal side of the cyst while the basal bodies and nascent flagellar axonemes localize to the opposite side. The flagellar axonemes then begin elongating towards the mitotic spermatogonia and stem cells at the apical end of the testis (Fig. 1A). At the tip of elongating tails are ring canals (that function to connect the 64 cells in the cyst) and a ciliary sheath that encases the bundled elongating flagellar axonemes. The remainder of the axoneme is ensheathed by mitochondria and a plasma membrane. Once the spermatids are fully elongated, the process of individualization begins. A special actin based structure, called the individualization complex (IC), forms from the mature spermatid nuclei, and then translocates down the axonemes, remodeling the membranes to separate the individual sperm tails and removing excess cytoplasm [12]–[16].

One of the critical steps in sperm differentiation is the polarization of the 64-cell spermatid cyst. There are multiple levels of polarization: At the cellular level, the round, seemingly symmetric spermatids must become polarized so that their nuclei are localized on one side, while the nascent flagellar initiate their growth on the opposite side. At the next level, polarization of individual spermatids in one cyst must be coordinated so that the all 64 of the spermatids in the cyst have the same orientation, nuclei clustered on one side of the cyst and nascent flagellar axonemes on the other. The two somatic cells that surround the cyst are also polarized: the head cyst cell encapsulates the clustered spermatid nuclei while the tail cyst cell covers the growing flagellar axonemes. During the following spermatid elongation and differentiation stages, the two somatic cells expand their volumes without divisions and retain their relative positions. Finally, the elongating spermatids must be oriented correctly with respect to the apical basal axis of the testes so that once differentiation is complete the mature sperm can be readily transferred into the seminal vesicle.

Here we show that aPKC is required to orient the direction of spermatid cyst polarization with respect the apical-basal axis of the testis. When *apkc* activity is compromised, the direction of polarization appears to be randomized. As in many other biological contexts, aPKC protein is asymmetrically localized to the apical side of the spermatid cyst during polarization. However, the mechanisms used to spatially restrict aPKC accumulation are different from the canonical cross-regulatory interactions between this kinase and other cell polarity proteins that normally orchestrate polarization. Instead, an mRNA localization pathway that is regulated by one of the two *Drosophila* CPEB family translational regulators, *orb2*, is responsible for spatially restricting the accumulation of aPKC protein during cyst polarization. We show that Orb2 binds to the 3′ UTR of a special *apkc* mRNA species, *apkc-RA*, which is only expressed in post-meiotic cysts, and regulates both its localization and translation. We also find that *orb2* functions not only in orienting cyst polarization with respect to the apical-basal axis of the testis, but also in the process of polarization itself. One of the *orb2* regulatory targets during polarization is its own mRNA, and this autoregulatory activity depends, in turn, upon *apkc*.

## Results

### aPKC is localized near the tips of spermatid flagellar axonemes

Although substantial amounts of aPKC can be detected in Western blots of testis extracts (not shown), much of this protein is likely to be somatically derived. In whole mounts of wild type testes probed with aPKC antibody, only a low level of unlocalized protein is detected in spermatocytes before and during the first and second meiotic divisions (Fig. 1B, arrowhead and arrow). However, after meiosis is complete and the 64-cell cysts show the first signs of polarization and axoneme nucleation, prominent aPKC signals can be detected. As shown in a newly polarized cyst in Fig. 1C, aPKC concentrates in “puncta” (arrow) that are clustered in a region of the cyst diametrically opposite to the spermatid nuclei. This region contains the nascent flagellar axonemes and it will form the tip of the elongating spermatid tails. As elongation proceeds and the structure of the elongating flagellar axonemes becomes more tightly organized, there is a band of aPKC protein located near the tip of the elongating sperm tails (Fig. 1D, arrow, insert). During elongation, the protein appears to be arranged into a series of thin stripes that run parallel to the bundled sperm tails and are located near if not at the termini of the 64 growing flagellar axonemes (Fig. 1E, arrow). These stripes remain associated with the tips of the elongated flagellar axonemes until after individualization commences (not shown).

In other biological contexts, proteins that function together with aPKC in cell polarization often exhibit similar or reciprocal localization patterns. To identify other potential players, we examined the expression of other classic polarity regulators Bazooka (Baz: *Drosophila* Par3), Discs large (Dlg) and Par-1 during spermatogenesis. As shown in Figure S1, high levels of Baz, Dlg and Par-1 were seen at the apical end of the testis, in the region that contains the germline stem cells and the mitotic spermatogonia/spermatocytes and the somatic support cells. These proteins seemed to be mostly expressed in the somatic support cells that surround the developing mitotic and meiotic germline cysts (Fig. S1A, C, E). However, unlike aPKC none showed localized accumulation later on during the differentiation of the spermatid cysts (Fig. S1B, D, F).

### *apkc* is required for cyst polarization

**Figure 2:**
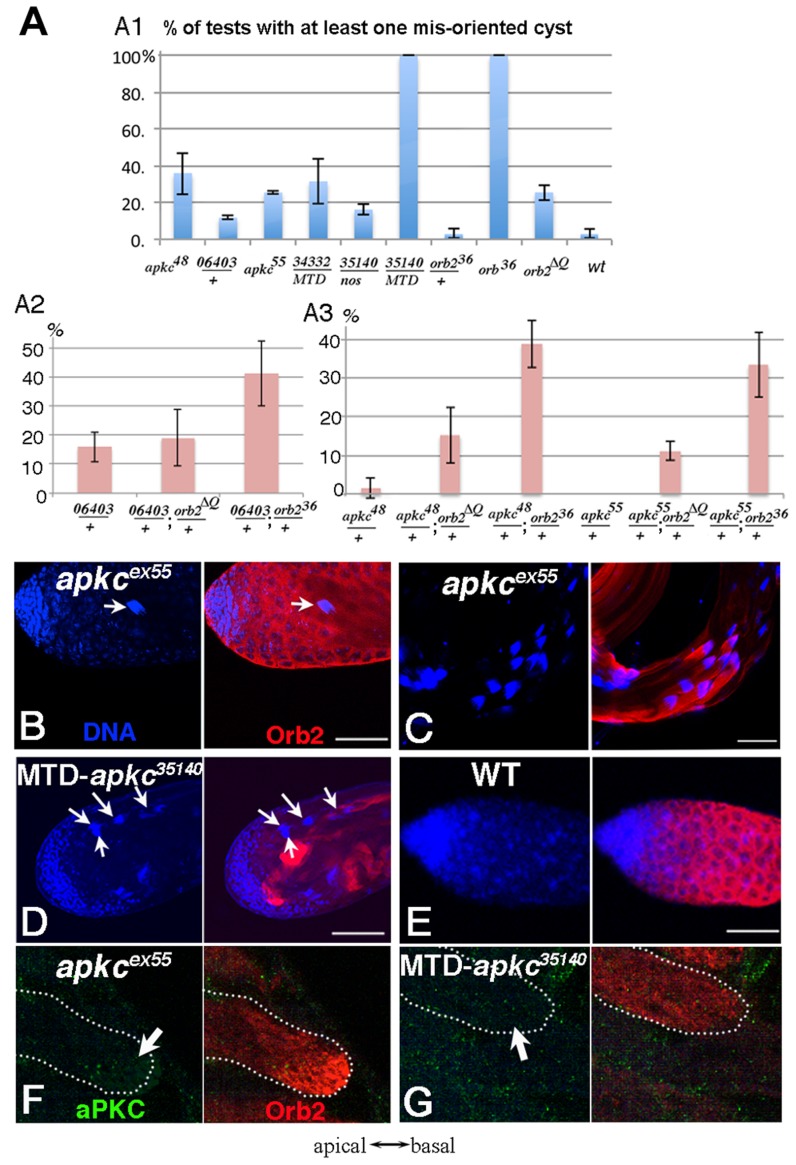
Spermatid cysts elongate in the wrong direction in *apkc* or *orb2* mutants. A) Frequency of testes that have at least one spermatid cyst polarized in the wrong orientation so that clustered spermatid cyst nuclei are found in the apical region instead of towards the base of the testis. *apkc^48^* (*apkc^ex48^*), *06403* (*apkc^06403^*) and *apkc^55^* (*apkc^ex55^*) are *apkc* mutant alleles. *34332* and *35140* are UAS-*apkc* RNAi lines (dsRNA targeting sites marked in Fig. 3A), while the Gal4 drivers are *nos* or MTD. MTD has three GAL4 drivers, *pCOG-Gal4* which has an *otu* promoter, plus two different *nos* drivers, *NGT-40* and *nanos*-*Gal4*. Error bars are standard deviation based on three experiments. Total number of testes scored for each genotype are (from left to right): Fig. A1: *apkc^ex48^*: 36; *apkc^06403^/+*:33; *apkc^ex55^*:27; MTD/UAS-*apkc:34332*:43; *nos-GAL4/*UAS-*apkc:35140*: 18; MTD/UAS-*apkc:35140*:16; *orb^36^/+*:55; *orb^36^*: 50; *orb2^ΔQ^*: 86; WT: 65. Fig. A2: *apkc^06403^/+*: 38; *apkc^06403^/*+; *orb2^ΔQ^*/+:40; *apkc^06403^/*+; *orb2^36^*/+: 49; Fig. A3: *apkc^ex48^/+*: 44; *apkc^ex48^/*+; *orb2^ΔQ^*/+: 39; *apkc^ex48^/*+; *orb2^36^*/+: 39; *apkc^ex55^/*+: 27; *apkc^ex55^/*+; *orb2^ΔQ^*/+: 28; *apkc^ex55^/*+; *orb2^36^*/+: 37. B-D) Whole mount antibody staining of wild type or *apkc* mutant testes. Blue, DNA; red, Orb2. B) Spermatid nuclei clusters (arrow) are found in the spermatogonial region of the testis in *apkc* hypomorphic alleles. The example shown here is *apkc^ex55^*. D) In the hypomorphic alleles, most spermatid nuclei clusters are located as in wild type on the basal side of testes. D) In MTD/UAS-*apkc:35140* testes, multiple spermatid nuclei clusters are found in the apical region (arrows). (Not all spermatid cysts express Orb2 and/or are in focus). E) In wild type testes spermatid cyst nuclei are never seen at the apical end of the testis. F) aPKC (green) and Orb2 (red) in a misoriented elongating *apkc^ex55^* spermatid cyst. Note that the Orb2 comet head is present. G) aPKC (green) and Orb2 (red) in a misoriented elongating MTD/UAS-*apkc:35140* testis. Note that the Orb2 comet is absent. This is also the case for correctly oriented MTD/UAS-*apkc:35140* spermatid cysts (not shown). All images are orientated with apical side of the testes to the left and basal to the right. Scale bars: 50 µm.

**Figure 3:**
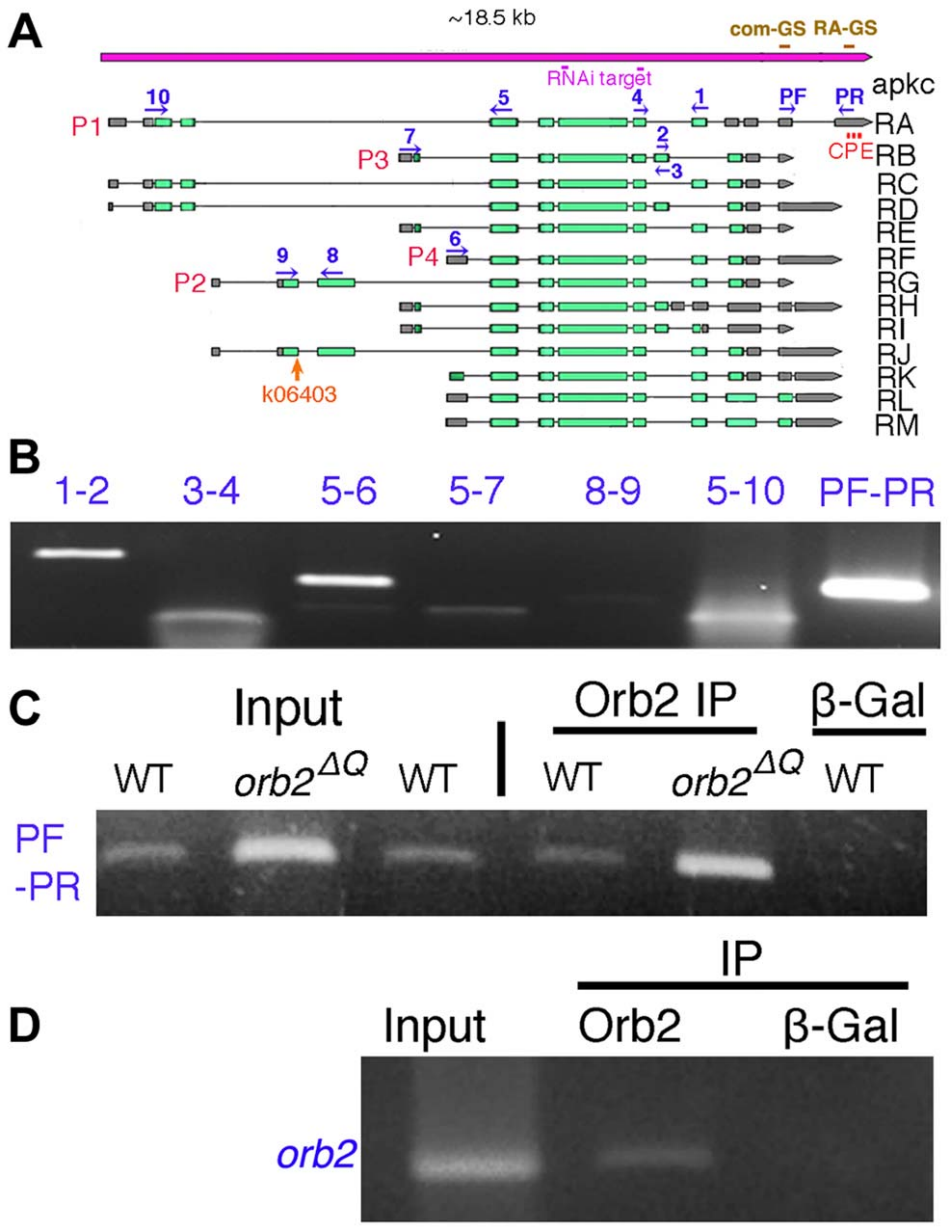
*apkc-RA* and *orb2* mRNAs associate with Orb2 *in vivo*. A) The *apkc* transcription unit as annotated in http://flybase.org/reports/FBgn0261854.html. Transcripts expressed from four *apkc* promoters (P1–P4) plus a collection of alternatively spliced exons and UTRs are predicted to generate more than ten mRNAs. Green bar, protein coding exon; grey bar, UTR; solid black line, intron. *apkc-RA* CPE sites are labeled in red. Positions of primers used for RT-PCR experiments to identify different *apkc* mRNA species are marked in dark blue. The two dsRNA sequences used in the *apkc* RNAi knockdown experiments are marked in pink. Probes for the *apkc* Orb2 EMSAs, com-GS and RA-GS, are indicated above the gene in brown. B) *apkc* transcripts expressed in testes were detected by RT-PCR using the primer pairs as indicated. Not all of the primer pairs are specific to one of the four promoters, but some are specific to particular splice forms. C) *apkc-RA* mRNA can be immunoprecipitated with Orb2 antibodies from WT and *orb2^ΔQ^* testis extracts. β-Gal antibody (a negative control) didn't immunoprecipitate *apkc-RA* mRNA. D) Orb2 antibody immunoprecipitated *orb2* mRNA from wild type testes while the control β-Gal antibody did not.

In many systems a critical step in the polarization pathway is the asymmetric localization of aPKC. Thus, a reasonable expectation is that the targeting of aPKC to the apical side of differentiating spermatid cysts will orchestrate some aspect(s) of cyst polarization. To explore this possibility we analyzed the effects of *apkc* mutations and RNAi knockdowns on sperm morphogenesis (Fig. 2). *apkc^k06403^* has a P-element insertion between the P2 and P3 promoters that could potentially affect mRNAs produced by the upstream P1 and P2 promoters (see map in Fig.3A). Since this allele is homozygous lethal, we looked for phenotypic effects in heterozygous mutant males. We also examined two viable hypomorphic alleles *apkc^ex55^* and *apkc^ex48^* that were generated by the excision of the *apkc^k06403^* P element [17] [18].

In wild type testes, the orientation of spermtid elongation is invariant: The sperm tails always extend towards the apical end of the testis while the sperm nuclei are pushed towards the base of the testis. As a result, spermatid nuclei clusters are not seen in the spermatogonia/spermatocyte region of the testis (Fig. 2E). However, when *apkc* activity is compromised, spermatid nuclei clusters appear at the apical side of the testes (Fig. 2B). Fig. 2A shows the frequency of testes that have differentiating sperm oriented in the opposite direction – the spermatid nuclei cluster located apically and the tails elongating basally. Whereas misoriented cysts were rarely if ever seen in wild type, about ¼ of the testes in males homozygous for either of the hypomorphic alleles had misoriented spermatid cysts. Within these testes, 5–20% of the cysts elongated in the incorrect direction. Arguing against simple background effects, we found that *apkc* was weakly haploinsufficient and incorrectly oriented spermatid cysts were also detected in males heterozygous for the strong loss of function allele, *apkc^k06403^*. It is worth noting that unlike other genes that have been implicated in cyst polarization (e.g., the exocyst complex: [19]), the defect in these hypomorphic *apkc* mutants appears to be in choosing the direction of polarization and not in coordinating or executing the polarization process itself. This is suggested by the fact that all 64 of the spermatids in the mutant cysts undergo seemingly normal elongation but in the incorrect direction and we never observed spermatids within the same cyst elongating in opposite directions.

To provide further evidence that *apkc* is required to properly orient cyst polarization within the testis and also determine whether it functions in the germline, we knocked down *apkc* by combining *UAS-apkc* RNAi lines with a germline specific Gal4 driver *nanos*-*Gal4*, or a triple Gal4 line (*pCOG-Gal4*; *NGT-40*; *nanos*-*Gal4*), MTD, that drives higher levels of UAS dependent expression in germline cells in most stages of spermatogenesis [20], [21]. To control for off target effects from RNA interference, we tested two *UAS*-*apkc* RNAi lines, *UAS-apkc::34332* and *apkc:35140*, that express double strand RNA, dsRNA-HMS01320 and dsRNA-GL00007, targeting two different exon regions that are common for all *apkc* mRNA species (Fig. 3A) (see materials and methods) [22].

Orientation defects like those observed in the hypomorphic mutants were also seen when *apkc* activity was reduced by RNAi knockdown (Fig. 2D). For example, at 22o C 18% of the *nanos*-Gal4/UAS-*apkc:35140* testes had at least one incorrectly oriented spermatid cyst (Fig. 2A). Moreover, amongst the testes that had polarity defects, nearly 20% of the spermatid cysts were elongating in the wrong direction. The frequency of *nanos*-Gal4/UAS-*apkc:35140* testes with incorrectly oriented elongating spermatid cysts increased from 18% to 45% when the temperature was raised from 22o C to 25o C. Even stronger effects were observed when UAS-*apkc:35140* was combined with the triple Gal4 line MTD. In this case, every testis had multiple misoriented spermatid cysts (Fig. 2A). In addition to these orientation defects, the spermatids in testes from the MTD/UAS-*apkc:35140* knockdown were shorter than wild type (not shown). This defect suggests that *apkc* activity might be needed to sustain the elongation of the flagellar axoneme.

### Post-meiotic expression and localization of *apkc-RA*

Since several of the proteins that usually collaborate with aPKC do not seem to be expressed in differentiating spermatids, other mechanisms are likely important for localizing aPKC asymmetrically and facilitating its function in correctly orienting cyst polarization. One such mechanism would be mRNA localization. Support for this possibility comes from the studies of Barreau *et al.* [23]. They identified a special group of genes that are transcribed only post-meiotically and encode mRNAs that localize in a striking “comet” pattern at the apical end of the elongating spermatid tails. The “head” of the comet, where these mRNAs are most highly concentrated, is in the same region of the elongating tails as the aPKC protein stripes.

**Figure 4:**
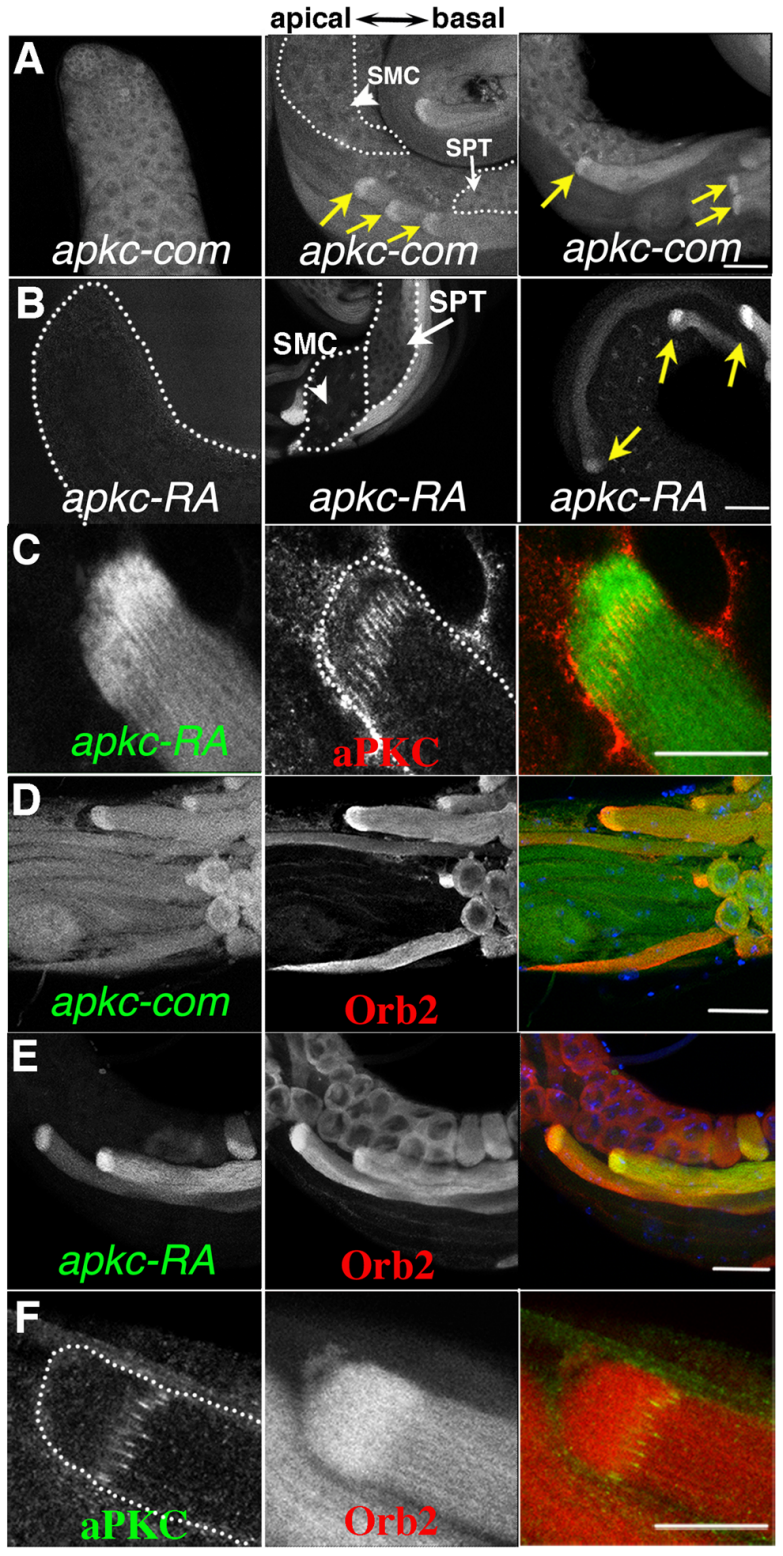
Localized accumulation of *apkc* mRNA, aPKC and Orb2 protein. A, B) Testes were probed with *apkc-com* or *apkc-RA* specific fluorescent oligos. Panels highlight *apkc* expression at different stages of spermatogenesis. A) Left panel: mRNAs complementary to the *apkc-com* mRNA probe (specific for an exon that is present in all of the predicted *apkc* mRNA species – “bulk” *apkc* mRNA) were detected in the stem cell/spermatogonial/early spermatocytes region of the testis. Middle and right panel: “Bulk” *apkc* mRNA complementary to the *apkc-com* probe is also present at moderate levels in spermatocytes (SMC) and spermatids (SPT) (white arrows). In elongating spermatid cysts bulk *apkc* mRNA is localized in a comet pattern (yellow arrow). B) Left panel: Unlike “bulk” *apkc* mRNA, *apkc-RA* is not expressed in stem cells or spermatogonial cysts. Middle panel: *apkc-RA* is also absent in spermatocytes (SMC, arrowhead). Low levels of *apkc-RA* are first detected in spermatid cysts (SPT, white arrow) after meiosis is complete but prior to cyst polarization. Right panel: High levels of *apkc-RA* are present during the elongation phase (yellow arrow). At this stage *apkc-RA* accumulates in the characteristic comet pattern. C) aPKC protein stripes are found at the proximal (away from the elongating frontier) edge of the *apkc-RA* comet head. D) “Bulk” *apkc* mRNAs (detected with the *apkc*-*com* probe) and E) *apkc-RA* mRNAs co-localize with Orb2 in a comet pattern. The most extensive co-localization of mRNA and protein appears to be in the comet head. F) aPKC protein stripes accumulate at the proximal edge of the Orb2 protein comet head. All images are orientated with the apical end of the testis is to the left and basal end to the right. Scale bar: 20 µm.

To determine if *apkc* mRNA is similarly localized in differentiating spermatids, we generated a set of oligo probes specific to the large exon that is common to all *apkc* mRNAs (Fig. 3A). *apkc* mRNAs can first be detected in the stem cell region of the testis and they persist until late in spermatogenesis (Fig. 4A). Significantly, in differentiating spermatid cysts *apkc* mRNAs are distributed in a “comet” pattern just like the post-meiotically expressed mRNAs identified by Barreau *et al*. (Fig. 4A, arrow). The highest concentration of *apkc* mRNA is in the comet “head” which is close to the leading edge of the elongating sperm tails, while there is a comet “tail” with lower levels that extends backwards towards the spermatid nuclei.

The *apkc* gene has four different promoters (P1–P4) and, together with an extensive set of alternatively spliced exons and UTRs, it is predicted to generate more than 10 different mRNAs (Fig. 3A). To identify *apkc* mRNA species that are expressed in the testis we used primers specific for different promoters and the alternatively spliced exons and UTRs (e.g., 1-2/3-4/PF-PR) for RT-PCR (Fig. 3A, 3B). These experiments indicate that testes have a complex mixture of many *apkc* mRNA species. Interestingly, one of these mRNAs, RA (P1: 5–10; PF-PR), has an unusually long 3′ UTR that is not found in any of the other predicted mRNA species.

As mRNA localization often depends upon special *cis*-acting elements in the 3′ UTR, we generated *in situ* probes specific for *apkc-RA* mRNA species. The *apkc-RA* expression pattern differs substantially from that of the “bulk” *apkc* mRNAs that hybridizes to the large common exon probe. Unlike “bulk” *apkc* mRNA, *apkc-RA* can not be detected in the pre-meiotic stages, while only low levels are present in the meiotic cysts (Fig. 4B, arrow head & Fig. S2A). Its expression is first detected in the round spermatids after meiosis is complete (Fig. 4B, white arrow), and increases to a maximum during spermatid differentiation (Fig. 4B, yellow arrows & Fig. S2A). Initially *apkc-RA* is distributed uniformly through the cyst; however, this changes when polarization commences and the spermatid nuclei start to cluster on the basal side of the cyst. At this point *apkc-RA* mRNAs can be detected near the apical tip of the nascent flagellar axonemes much like aPKC protein (Fig. S2B). As elongation proceeds, *apkc-RA* mRNAs accumulate in the characteristic comet pattern, and are most highly concentrated in the comet head near the apical tip of the elongating tails (Fig 4B, yellow arrows).

The *apkc-RA* mRNAs in the comet head are expected to be in close proximity to the aPKC protein stripes that mark the ends of the flagellar axonemes. When we simultaneously probed testes for *apkc-RA* mRNA and aPKC protein, the aPKC stripes partially overlap with the proximal side of the *apkc-RA* mRNA comet head (Fig. 4C). This finding would fit with the idea that the *apkc-RA* mRNAs in the comet head are a source of the aPKC stripes at the leading edge of the growing flagellar axonemes.

### Orb2 binds to *apkc-RA* mRNA

Several of the post-meiotic mRNAs identified by Barreau *et al.* have consensus CPEs (Cytoplasmic Polyadenylation Elements) in their 3′ UTRs. CPE motifs are recognized by a conserved family of RNA binding proteins, CPEBs, which in flies are known to function both in mRNA localization and translational regulation [24]–[26]. Proteins in this family have two RRM-type RNA binding domains in their C-terminal halves, while their N-terminal halves contain polypeptide sequences that have regulatory functions [27], [28]. Inspection of the unique *apkc-RA* 3′ UTR indicates that it has three canonical CPE motifs (Fig. 3A and Text S2). Several of the other *apkc* mRNA species (RD, RJ, RK, RL and RM), which share a different UTR sequence, have a single CPE sequence. Thus, an intriguing idea is that a fly CPEB protein binds to the RA mRNA species and perhaps to one or more of these other *apkc* mRNAs, facilitates their localization to the tip of the elongating sperm tails and controls their translation. Flies have two CPEB genes, *orb* and *orb2*, and both are expressed in testes. Of these, *orb* is not likely to have either of these functions as its message is not translated until after spermatid elongation is complete [29]. *orb2*, on the other hand, would be a plausible candidate for the regulatory factor. It is required at multiple steps during spermatogenesis including spermatid differentiation. We've found that one of its differentiation functions is to control the translation of post-meiotically expressed mRNAs like *orb* that have CPE motifs in their 3′ UTRs. Moreover, like these post-meiotic mRNAs, Orb2 protein is distributed in a comet pattern in elongating spermatids [29]. Two other findings lend support to the idea that *orb2* might regulate the localization and/or translation of *apkc-RA* and perhaps other CPE containing *apkc* mRNAs species in the testis. First, we found that *orb2* is required for the apical accumulation of aPKC in embryonic neuroblasts during asymmetric cell division [30]. Second, exogenous biotin tagged *apkc-RA* RNA can pull down Orb2 protein from adult fly brain extracts [31]. This hypothesis makes several predictions that we have tested for the *apkc-RA* mRNA species as it has a post-meiotic expression pattern.

#### Recombinant Orb2 should bind to the RA 3′ UTR in vitro

To test this prediction, we used recombinant Orb2 to gel shift a ∼150 nucleotide probe containing one of the CPE sequences in the RA 3′UTR (RA-GS in Fig. 3A) and a similar sized probe from a 3′ UTR sequence that is present in all *apkc* mRNAs (com-GS in Fig. 3A) and doesn't have any CPE-like sequences (see Text S1). We found that recombinant Orb2 shifted the *RA* 3′ UTR (RA-GS) probe, but not the common 3′ UTR (com-GS) probe (Fig. S3 and Text S1, S2). We next generated two cold 24 nt competitor RNAs. One of these, Comp-CPE, spans the first CPE sequence in the *apkc-RA* 3′ UTR; while the other 24 nt oligo, Comp-com, was derived from com-GS sequence and didn't contain a CPE-like element (Text S1, S2). The shift of the *RA* 3′ UTR probe by recombinant Orb2 can be competed by excess cold Comp-CPE competitor (Fig. S3). In contrast, the Comp-com oligo, which doesn't have a CPE sequences competes poorly if at all. Equivalent results were obtained when the labeled probes were the 24 nt oligos and the cold competitors were the longer RNAs.

#### Orb2 should be associated with the RA 3′ UTR in vivo

We tested this prediction by immunoprecipitating testes extracts with Orb2 antibodies and then analyzing the RNA associated with Orb2 protein using RT-PCR. Orb2 antibodies immunoprecipitated *apkc-RA* mRNA (PF-PR) from testis extracts, while β-galactosidase antibodies did not (Fig. 3C and Text S1). In additional control experiments, *orb* plus several other CPE containing post-meiotic mRNAs were found in the Orb2 immunoprecipitates, while the negative control mRNA, *boule*, was not (data not shown: see [22]).

#### *apkc* mRNA and Orb2 should co-localize

If *apkc-RA* mRNA is bound by Orb2, it should co-localize with Orb2 in differentiating spermatids. Fig. 4D and E show that Orb2 is distributed in a comet pattern in elongating spermatids just like *apkc* mRNA. The merged image in Fig. 4E shows that much of *apkc-RA* mRNA in elongating spermatids co-localizes with Orb2, particularly in the region containing the Orb2/*apkc-RA* comet heads. Similar results are obtained for the “bulk” *apkc* mRNAs using the common exon probe (Fig. 4D) (though the relative enrichment in the comet head does not seem to be as great as that observed for *apkc-RA*). While Orb2 protein and *apkc-RA* mRNA disappear as soon as elongation is complete, *apkc* mRNA can still be detected with the common exon probe during the individualization phase (data not shown).

#### aPKC protein should localize near or in the Orb2 comet head

The aPKC protein stripes at the tips of the elongating flagellar axonemes are for the most part localized on the basal (proximal) side of the *apkc* mRNA comet head. If Orb2 controls the localized translation of *apkc* mRNAs in the comet head, then the aPKC protein stripes should overlap the Orb2 comet head. This is the case (Fig. 4F).

### Spermatid elongation defects in *orb2* mutants

The experiments in the previous section demonstrate that Orb2 co-localizes with *apkc* mRNAs in elongating spermatid cysts and binds directly to the 3′ UTR to an *apkc* mRNA species that is specifically expressed at this stage of spermatogenesis. If this association is important for *apkc* activity during spermatid differentiation, *orb2* should also be required for correctly polarizing the 64-cell cysts. To test this prediction we examined spermatid cyst polarization in two different *orb2* mutants, *orb2^36^* and *orb2^ΔQ^*. *orb2^36^* is a null allele that lacks the entire Orb2 protein coding sequence. Meiosis is blocked in *orb2^36^* and 16-cell cysts, which have duplicated their DNA but not undergone the 1^st^ meiotic division, accumulate in the mutant testes. These cysts ultimately exit meiosis and then attempt but fail to differentiate into mature sperm [29]. The other allele, *orb2^ΔQ^*, is a hypomorph. It has an in frame deletion that removes a short poly-glutamine domain in the N-terminal half of the protein. While the sequence of the Orb2 protein is altered, its level of expression remains the same [32]. Unlike the null, meiosis is unaffected in *orb2^ΔQ^*, and *orb2^ΔQ^* males produce some functional sperm and are fertile. However, in a subset of the spermatid cysts there are abnormalities in spermatid differentiation, including polarization defects (Fig. 2A). The polarization phenotype in this hypomorphic *orb2* allele closely resembles that seen for *apkc*, and we will discuss it first.

**Figure 5:**
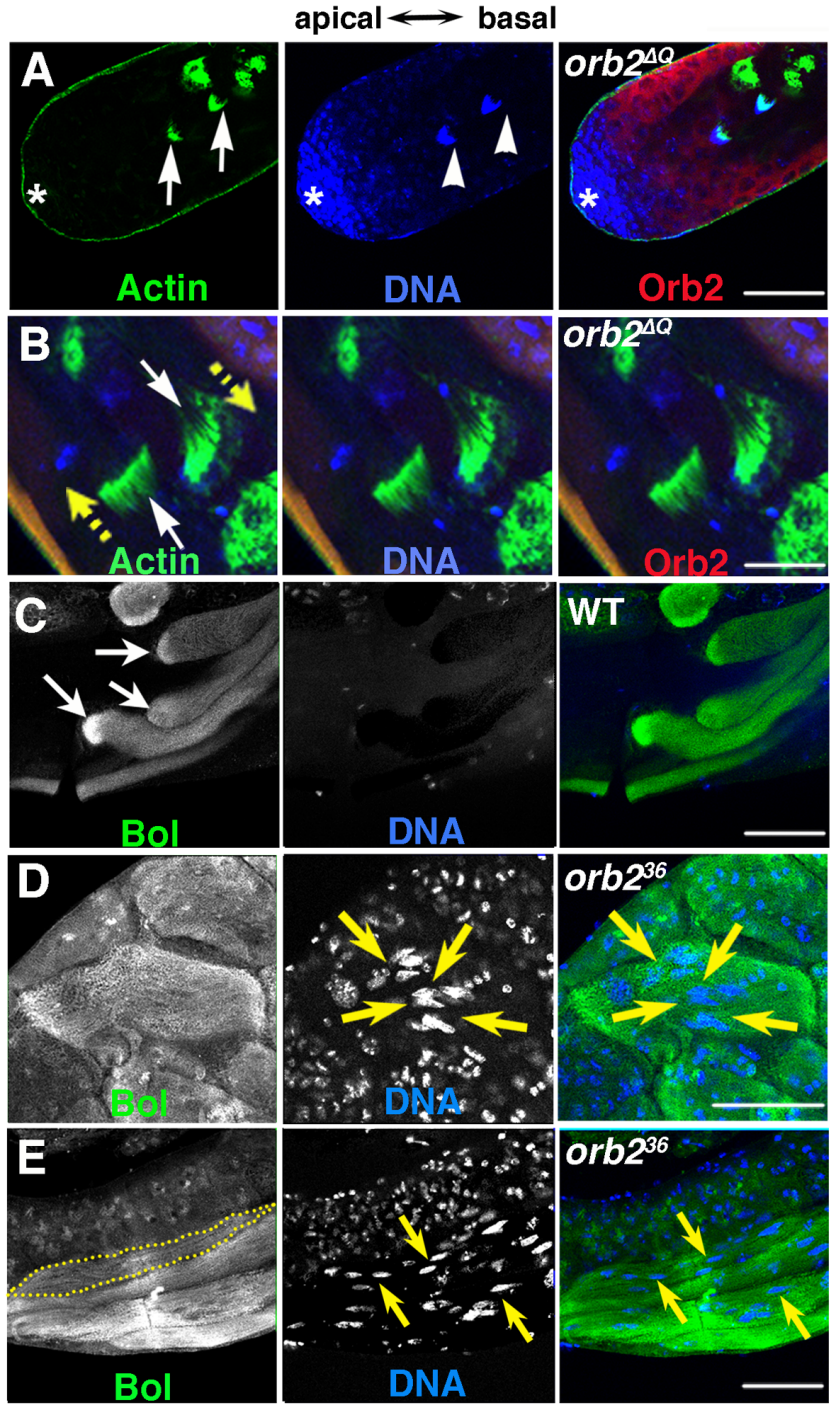
*orb2* is required for cyst polarization. A and B) Green, phalloidin labeled Actin; Blue, DNA; Red, Orb2. A) Clustered nuclei of spermatid cysts (arrowheads) that have completed elongation and just assembled individualization complexes (ICs) (arrows) are found in the spermatogonial/early spermatocyte region of *orb2^ΔQ^* testis. B) White arrows point to two ICs in an *orb2^ΔQ^* testis that are moving in opposite directions in the middle of the testis (yellow arrows indicate directions of motion). C–E) Green, Bol; blue, DNA. C) Like Orb2, the translation factor Bol is localized a comet pattern during flagellar axoneme elongation in wild type testes [29]. D) A bi-polar *orb2^36^* spermatid cyst that has Bol concentrated at both elongating ends, while the nuclei are in the middle of the cyst (arrows). E) A partially elongated *orb2^36^* spermatid cyst in which spermatid nuclei and Bol protein are scattered randomly. Dotted line outlines one among four spermatid cysts in the figure. Yellow arrows point out a few of the scattered nuclei. All images are orientated with apical side of the testes to the left and basal to the right. Scale bar: 50 µm.

One of the differentiation defects in *orb2^ΔQ^* is in properly orienting the direction of spermatid cyst polarization. This phenotype is observed in slightly over 20% of the *orb2^ΔQ^* testes (Fig. 2). Fig 5A and B show spermatid cyst nuclei and individualization complexes (IC) in *orb2^ΔQ^* testes visualized by Hoechst staining of DNA (blue, arrowheads) and phalloidin staining of actin (green, arrows). The IC consists of 64 aligned actin cones. After elongation is complete, the IC assembles using the condensed spermatid nuclei as a scaffold and then travels along the flagellar axonemes separating syncytial spermatids from each other. The *orb2^ΔQ^* testes in Fig. 5A has two assembled ICs (actin in green) that are still associated with the condensed spermatid nuclei (DNA in blue); however, instead of being located towards the basal end of the testis as in wild type, the two spermatid nuclei clusters and associated ICs are in the spermatocyte region of the testis (“*” marks stem cell position). ICs have a distinct morphology; the flat side faces the direction of IC motion, while the thinner, rounded side faces the cluster of spermatid nuclei. In *orb2^ΔQ^* testes, ICs moving in opposite directions were sometimes observed (Fig 5B, yellow arrows indicate moving directions).

While *orb2^ΔQ^* has defects in choosing the direction of cyst polarization with respect to the apical-basal axis of the testis, the spermatids within the cyst undergo a coordinated (all in the same direction) and otherwise seemingly normal polarization. In contrast, as illustrated by the disorganized distribution of the Boule translation factor and spermatid nuclei, the process of cyst polarization itself seems to be disrupted in *orb2^36^*. In wild type, Boule accumulates in a comet pattern near the tip of the elongating flagellar tails just like Orb2 (Fig. 5C, arrows) [29], [33]. In the improperly polarized *orb2^36^* cyst in Fig. 5D), there are Boule “pseudo-comets” extending from both ends of the cyst towards the spermatid nuclei, which are scattered near the center of the cyst (arrows). In the partially elongated cyst shown in 5E, Boule is distributed along the apical-basal axis with little evidence of polarization, while the spermatid nuclei are scattered throughout much of the cyst. Although these findings indicate that *orb2* is essential for cyst polarization, the possibility remains open that the polarization defects arise at least in part from the block in meiosis.

### Genetic interactions between *orb2* and *apkc*

If the failure to properly orient the direction of spermatid polarization in *orb2^ΔQ^* arises because *apkc* requires *orb2* activity in this process, we might expect to observe genetic interactions between these two genes. To explore this possibility we examined spermatid cyst polarization in different *orb2/apkc trans*-heterozygous mutant combinations. As described above, *apkc* is weakly haploinsufficient in cyst polarization, and in flies heterozygous for the strong loss of function allele *apkc^k06403^* about 15% of the testes had improperly oriented spermatid cysts (Fig. 2A2). By contrast, essentially no polarization defects were evident in testes from flies heterozygous for either *orb2^ΔQ^* (not shown) or *orb2^36^* (Fig. 2A1). On the other hand, about 40% of the testes from *orb2^36^*/*apkc^k06403^ trans*-heterozygous males have misoriented spermatid cysts (Fig. 2A2). While *orb2^ΔQ^* has at most only a minimal effect on the number of testes that have incorrectly oriented spermatid cysts when combined with *apkc^k06403^*, this allele significantly increases the frequency of cyst polarization defects when combined with the weak loss of function *apkc* mutants *apkc^ex48^* and *apkc^ex55^*(Fig. 2A3). In both *trans*-heterozygous combinations, the frequency of testes with misoriented spermatid cysts increases more than 10-fold. An even more dramatic genetic interaction is observed when the *orb2* null allele, *orb2^36^*, is combined with either *apkc^ex48^* or *apkc^ex55^* (Fig. 2A3). In fact, the frequency of testes with misoriented cysts in these two *trans*-heterozygous combinations is equivalent to that observed in the testes of the corresponding homozygous *apkc* mutant.

### *orb2* is required for localizing and translating *apkc* mRNAs during elongation

**Figure 6:**
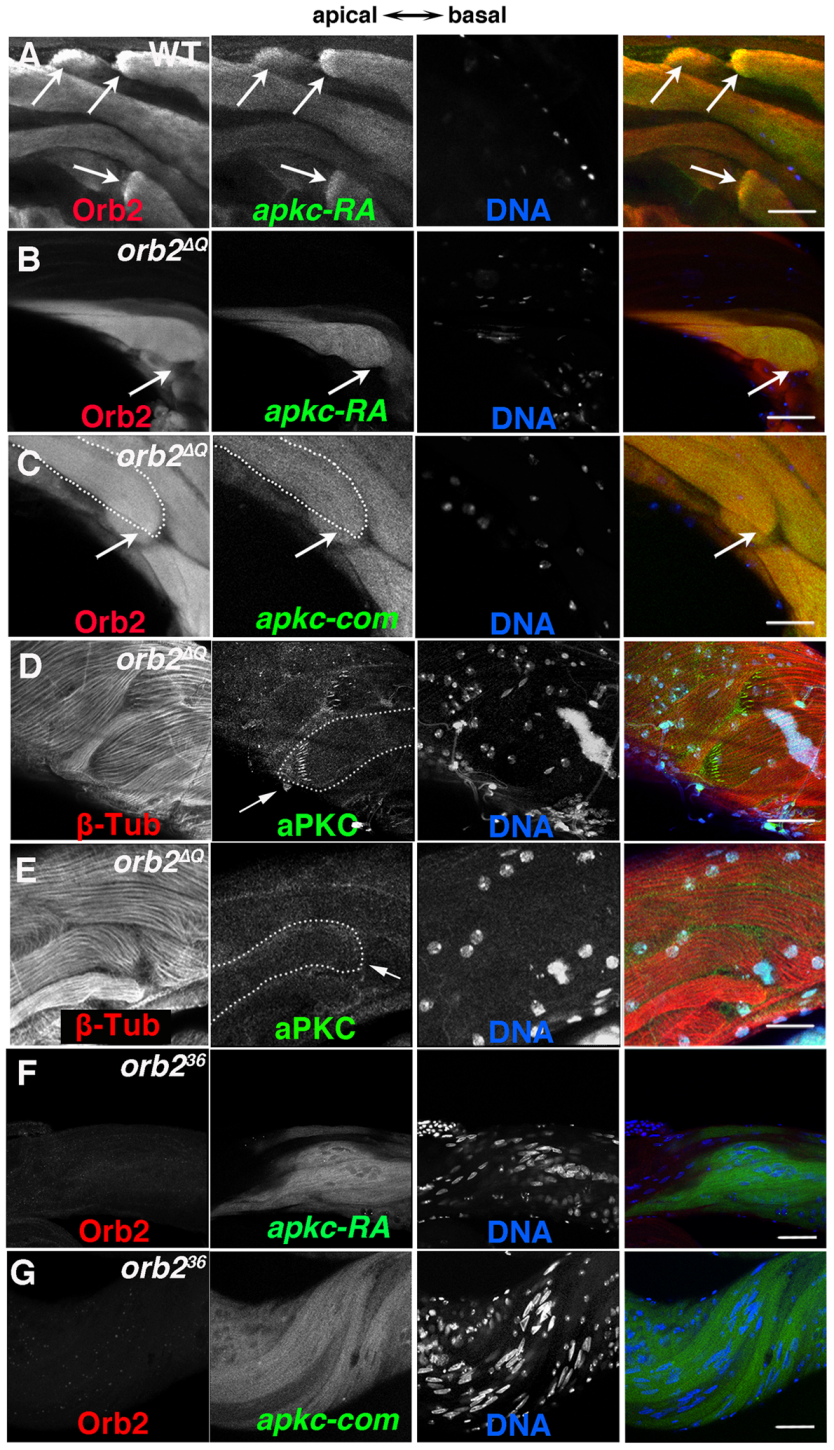
*orb2* is required for polarized accumulation of *apkc-RA* mRNA and aPKC protein in spermatid cysts. A) In wild type testes, Orb2 and *apkc-RA* accumulate in a comet pattern in elongating spermatid cysts located towards the apical side of the testis. B and C) In *orb2^ΔQ^* spermatid cysts that elongate in the incorrect direction, Orb2^ΔQ^ protein and both *apkc-RA* mRNA (detected with the *apkc-RA* probe, B) and “bulk” *apkc* mRNA (detected with the *apkc-com* probe, C) are distributed randomly instead of localizing in the comet pattern. Arrows in A indicate comet heads. In B and C the arrows indicate the expected position of the comet head. In the merged images on the far right, Red: Orb2; Green: *apkc* mRNA; Blue: DNA. D & E) aPKC protein (arrow) in *orb2^ΔQ^* spermatids elongating in the correct (D) or incorrect (E) orientation. *orb2^ΔQ^* spermatids elongating in the incorrect direction lack aPKC protein stripes at the tip of the elongating flagellar axonemes (compare arrows in D and E). In the merged images on the far right, Red: Tublin; Green: aPKC; Blue: DNA. F & G) *apkc-RA* (detected with the *apkc-RA* probe) and “bulk” *apkc* mRNAs (detected with the *apkc-com* probe) are expressed in *orb2^36^* spermatids but are not localized. In the merged image on right, Red: Orb2, Green: *apkc-RA* or bulk (*apkc-com*) mRNA, and Blue: DNA. All images are orientated with apical side of the testes to the left and basal to the right. Scale bar in A–D: 20 µm; in E and F: 50 µm.

These synergistic genetic interactions support the idea that the functioning of *apkc* in orienting spermatid cyst polarization within the testis depends upon *orb2*. An expectation of this hypothesis is that there should be defects in the targeting of *apkc* mRNA and/or in the localized expression of the aPKC protein when *orb2* activity is compromised. We first examined the localization of *apkc-RA* and “bulk” *apkc* mRNAs in *orb2^ΔQ^*. In *orb2^ΔQ^* cysts that were polarized in the incorrect direction, the comet pattern was invariably lost and *apkc-RA* mRNA was instead distributed almost uniformly along the length of the flagellar axonemes (Fig. 6A, B). Similar results were seen when we used the exon probe to visualize the “bulk” *apkc* mRNA (Fig 6C) indicating that the localization of other *apkc* mRNA species in the comet pattern is disrupted in incorrectly polarized cysts. On the other hand, in most, but not all of the *orb2^ΔQ^* cysts that polarized correctly and have their axonemes elongating towards the apical end of the testis, *apkc-RA* and the “bulk” *apkc* mRNAs were localized in the characteristic comet pattern as in wild type (not shown). The pattern of aPKC protein accumulation in *orb2^ΔQ^* parallels that of *apkc-RA* mRNA: aPKC stripes are absent in flagellar axonemes that are polarized in the wrong orientation (Fig.6E). In contrast, in most of the cysts that are polarized in the correct orientation, aPKC protein stripes are localized at the tips of elongating flagellar axonemes just like wild type (Fig.6D). Interestingly, *apkc-RA* mRNA is readily detected in Orb2 immunoprecipitates of extracts from *orb2^ΔQ^* testes (Fig. 3D). Thus, it seems likely that this deletion partially compromises a step subsequent to binding to the *apkc-RA* mRNA that is important for localizing the *apkc-RA;orb2* mRNP complex and/or activating translation.

We also examined *apkc* mRNA localization and translation in the null allele *orb2^36^*. In spite of the fact that meiosis never takes place, the *apkc-RA* transcript is still expressed in *orb2^36^* spermatid cysts. This was also true for *orb*, which, like *apkc-RA*, is transcribed post-meiotically in the germline of wild type males [29]. Fig. 6F and G show that the *apkc-RA* and also “bulk” *apkc* mRNAs are completely unlocalized in differentiating *orb2^36^* spermatid cysts. Likewise, the sharp stripes of aPKC protein seen near the ends of the growing sperm tails in wild type cysts are absent in the null mutant cysts (not shown).

### Orb2 appears to mediate the localization and translation of *orb2* mRNA

Fully extended flagellar axonemes are up to 1.8 mm in length [12], [13]. In order to mediate the localization and translational regulation of *apkc* or any other mRNA at the tips of the axonemes as the sperm tails elongate, there would have to be a continuous source of Orb2 in the comet head. One way to maintain high levels of Orb2 at the tip of the elongating axonemes would be a positive autoregulatory loop in which Orb2 helps direct the localization and translation of its own mRNA. An *orb2* autoregulatory activity could also help promote the initial polarization of the spermatid cyst. Moreover, there is precedence for CPEB proteins having autoregulatory activity. In the female germline, a two step positive autoregulatory mechanism, in which Orb mediates the localization and translation of *orb* mRNA, is known to be important in targeting Orb to the oocyte when it is first specified and then ensuring that Orb continues to accumulate within oocyte as it develops [34]. Also consistent with this idea, *orb2* mRNAs have consensus CPE motifs in their 3′ UTRs (Text S2).

**Figure 7:**
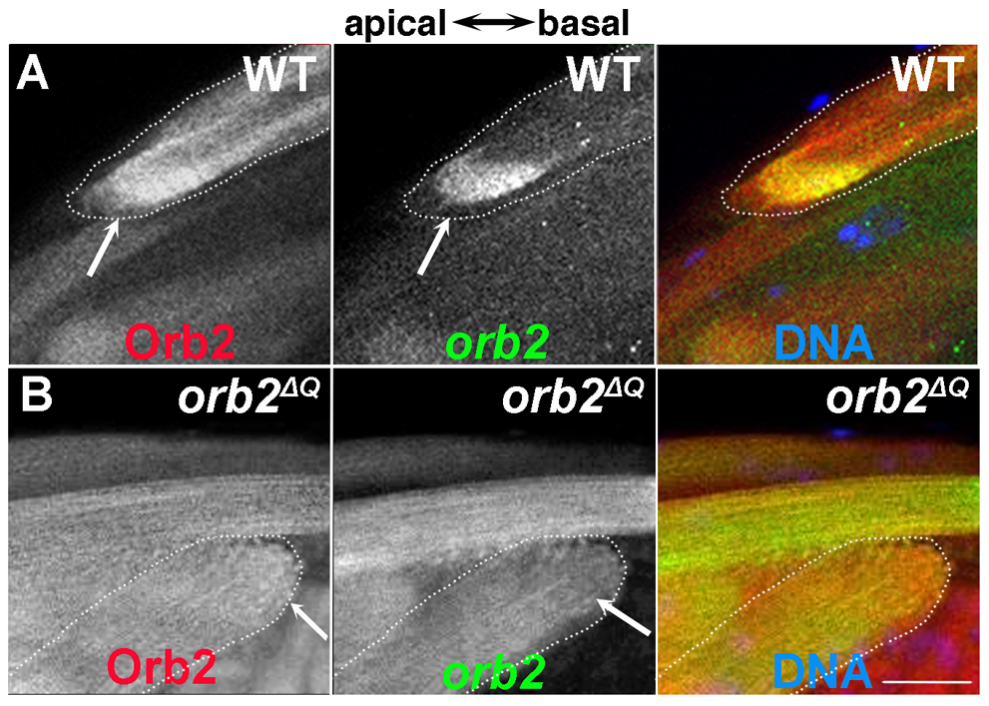
*orb2* autoregulates the localization and translation of *orb2* mRNA. A) Wild type spermatid cyst showing that Orb2 protein (red) and *orb2* mRNA (green) are distributed in the characteristic comet pattern and display extensive co-localization, particularly in the comet head. B) In *orb2^ΔQ^* spermatids that elongate in the incorrect direction, Orb2^ΔQ^ protein (red) and *orb2^ΔQ^* mRNA (green) are distributed uniformly in the cyst and there is no evidence of a comet head (expected position is indicated by arrow). All images are orientated with apical to the left and basal to the right. Scale bar: 20 µm.

To explore this possibility further we first asked if *orb2* mRNA is associated with Orb2 protein in testes extracts. We found that *orb2* mRNA can be immunoprecipitated with Orb2, but not control LacZ antibody (Fig. 3D). We next asked whether *orb2* mRNA and protein co-localize in elongating wild type and *orb2* mutant spermatids. As shown in Fig. 7A, there is generally a close correspondence between *orb2* mRNA and protein in elongating wild type spermatids. Importantly, both are concentrated and overlap extensively in the comet head.

Further evidence that *orb2* autoregulates the localization and translation of its own mRNA comes from analysis of *orb2^ΔQ^* testes. Just as was seen for *apkc* mRNAs and aPKC protein, the effects of *orb2^ΔQ^* depend upon the direction of polarization. In spermatids that are polarized in the incorrect orientation, *orb2* mRNA and Orb2 protein do not accumulate to high levels in the comet head at the end of the elongating flagellar axonemes. Instead, the mRNA and protein are distributed uniformly throughout the elongating sperm tails (Fig. 7B). In contrast, in most of the spermatids that are polarized in the correct direction, *orb2* mRNA and protein are localized in the characteristic comet pattern seen in wild type testes (not shown). The apparent failure of *orb2* autoregulation in *orb2^ΔQ^* cysts that are incorrectly polarized could also explain why *apkc* mRNAs are not localized in the comet pattern and why there are no aPKC protein stripes.

### *apkc* is required for the localized expression of Orb2 protein

One explanation for the strong *orb2-apkc* genetic interactions is that the functioning of the *orb2* autoregulatory loop at this stage of spermatogenesis could be intimately linked to *apkc* activity. To test this idea, we examined Orb2 protein expression in elongating spermatid cysts in testes from the *apkc^ex55^* allele and from the MTD/UAS-*apkc:35140* RNAi knockdown. There were no obvious effects on Orb2 expression in the hypomorphic allele *apkc^ex55^* in either incorrectly or correctly oriented elongating spermatid cysts. On the other hand, we found that the accumulation of Orb2 protein in the characteristic comet pattern at the tips of the elongating flagellar axonemes is disrupted when *apkc* is knockdown using the MTD/UAS-*apkc:35140* transgene combination (Fig. 2G). In this case, the Orb2 comet pattern was absent in cysts that were oriented correctly as well as incorrectly. This finding would be consistent with the idea that spermatid cyst polarity is completely randomized in the strong MTD/UAS-*apkc:35140* RNAi knockdown.

## Discussion

In order to produce functional sperm, the spermatid cyst must polarize so that all of the nuclei cluster together on one side of the cyst and the basal bodies and nascent flagellar axonemes on the other side. How the cyst orients with respect to the organ (testes) in which it resides is invariant: the nuclei cluster on the basal side of the cyst, while basal bodies localize on the opposite side of the cyst so that the axonemes can elongate apically towards the stem cell region of the testis. The factors involved in polarizing the cyst and in correctly orienting the polarized cyst within the testis are largely unknown. Here we have identified two genes that function to orient cyst polarization with respect to the apical-basal axis of the testis, *apkc* and *orb2*. While the involvement of *apkc* in determining polarity has been extensively documented in many biological contexts, the spatial restriction of *apkc* activity by a mechanism that involves localized mRNA translational regulation is by contrast rather unusual. We have found that compromising *apkc* and *orb2* activities can give rise to similar polarization phenotypes. In both instances, the system that orients cyst polarization relative to the apical-basal axis of the testes is disrupted and the sperm tails in a subset of the spermatid cysts elongate in the incorrect direction.

During the first steps of polarization all of the spermatid nuclei in the cyst congregate on the basal side, while aPKC protein accumulates in a series of puncta on the other, apical side, near the ends of the nascent flagellar axonemes. As elongation progresses, the aPKC puncta transform into a series of short stripes at the ends of the elongating axonemes. That this spatially restricted pattern of aPKC accumulation is not only mediated by *orb2* but is also likely to be relevant to properly orienting polarization with respect to the main axis of the testis is most clearly illustrated by the phenotypes in the hypomorphic allele, *orb2^ΔQ^*. All *orb2^ΔQ^* cysts that polarize in the incorrect orientation share a common set of defects. First, instead of being localized in the characteristic comet pattern, *apkc-RA* and “bulk” *apkc* mRNAs are spread uniformly though the cyst. Second, the aPKC protein stripes at the ends of the growing flagellar axonemes are absent. Third, while Orb2 protein and *orb2* mRNA can be detected in these cysts, neither is localized. This latter finding would provide an explanation for the defects in both *apkc* mRNA localization and the expression of aPKC protein. By contrast, most but not all of the *orb2^ΔQ^* cysts that elongate in the correct direction resemble wild type: *apkc* and *orb2* mRNAs and Orb2 protein are localized in the characteristic comet pattern, with aPKC stripes at the ends of the flagellar axonemes.

One likely *orb2* regulatory target is the *apkc-RA* mRNA. Although *apkc* mRNAs can be detected with a common exon probe in the adult male germline in all but the last stages of spermatogenesis, the *apkc-RA* mRNA is unusual in that it is expressed post-meiotically, and like other post-meiotic transcripts, it accumulates in a comet pattern in elongating spermatid cysts. This mRNA is transcribed from one of the four *apkc* promoters, and differs from the 12 other predicted *apkc* mRNA species in that it has a unique 3′ UTR. The RA 3′ UTR contain 3 CPE motifs and we've found that Orb2 binds to it both *in vitro* and *in vivo*. As is true for other mRNA targets of CPEB proteins, it is likely that *orb2* promotes the localized production of aPKC by directly activating the translation of the *apkc-RA* mRNAs in the comet head. Consistent with this idea, the stripes of aPKC at the ends of the elongating flagellar axonemes are localized on the distal (towards apical tip of the testes) side of the Orb2 protein/*apkc-RA* mRNA comet head. Several other *apkc* mRNA species share a different UTR sequence that also contains CPE motifs. While we haven't examined the expression of the mRNAs containing this specific UTR sequence, it is possible that one or more is expressed at this stage of spermatogenesis. Since the *apkc* mRNAs detected with the common exon probe localize in a comet pattern that is similar to *apkc-RA* and this localization is disrupted in *orb2* mutants, it would appear that if other *apkc* mRNA species are expressed at this stage of spermatogenesis they could also be *orb2* regulatory targets.

Significantly, the regulatory relationship between *orb2* and *apkc* seems to be reciprocal. The first suggestion of cross-regulation came from the synergistic genetic interactions seen in *trans*-heterozygous combinations between *orb2* and *apkc* mutants. Whereas polarization defects are rarely observed in the testes of males heterozygous for the hypomorphic *apkc^ex48^* or *apkc^ex55^* alleles, when they are combined in *trans* with the hypomorphic *orb2^ΔQ^* allele the frequency of testes with misoriented spermatid cysts increases by more than 10-fold. *Trans*-heterozygous genetic interactions between weak hypomorphic alleles are somewhat unusual, and raise the possibility that the two interacting genes are functionally interdependent. Consistent with a cross-regulatory connection, Orb2 is not localized in its characteristic comet pattern in elongating flagellar axonemes when *apkc* activity is knocked down in the MTD/UAS-*apkc:35140* combination. Instead it is uniformly distributed in the elongating axonomes. This finding indicates that the localized accumulation of Orb2 protein in differentiating spermatid cysts depends upon *apkc* activity. Since Orb2 binds to its own mRNA and appears to be required for both the localization and translation of this mRNA during spermatid cyst differentiation, a plausible hypothesis is that *apkc* is a component of the *orb2* positive autoregulatory loop. While *apkc* need not function directly, it is interesting to note that Orb2 has three (high stringency) predicted aPKC phosphorylation sites (Ser146, Ser273, and Ser446) [35], and all three are phosphorylated in Orb2 protein isolated from testes (unpublished data). Thus it is possible aPKC phosphorylation facilitates the localized translation of *orb2* mRNA, and in turn the localization and translation of *apkc* mRNAs, by phosphorylating Orb2 protein. In principle, this reciprocal regulatory relationship could help trigger the choice of orientation at the start of polarization and then serve to reinforce this choice.

While our finding would be consistent with a model in which aPKC activity is spatially restricted by a mechanism that depends upon *orb2* localizing and regulating the translation of *apkc* mRNAs, many important questions remain unanswered. One is whether *apkc* is required only for choosing the direction of cyst polarization within the testis, or if it also has a role in the process of polarization itself. Neither the *apkc* mutants nor the knockdowns are useful in answering this question as they certainly retain *apkc* activity. On the other hand, it seems likely that *apkc* is needed during the formation of the sperm tails since the elongating tails in the strongest RNAi knockdowns are considerably shorter than in wild type. As Orb2 protein accumulation is perturbed in this RNAi knockdown, one of the *apkc* functions during this phase of spermatogenesis is to maintain high levels of localized Orb2 protein.

A similar question can be asked about *orb2*. With the caveat that the *orb2^36^* cysts might have structural abnormalities arising from the failure to undergo meiosis, the phenotypes of this mutant argue that *orb2* is required for polarization *per se*. If the only *apkc* function in this process is orienting the direction of polarization, then *orb2* would have to have regulatory targets that control the actual process of polarization. In fact, there are several plausible candidates. Studies by Fabian *et. al.* [19] have shown that phosphatidylinositol 4,5-bisphosphate (PIP_2_) and components of the exocyst complex are required for cyst polarization. The exocyst complex also mediates plasma membrane addition during spermatid elongation and localizes around the tip of the growing sperm tails. mRNAs encoding 4 of the 8 exocyst complex subunits (*sec3*, *sec8*, *sec10*, and *sec15*) have CPE sequences in their 3′ UTRs, and their localization and/or translation could be regulated by *orb2*. Another possible *orb2* regulatory target in cyst polarization is *cdc42*. This small membrane anchored GTPase directs exocyst complex localization during ciliogenesis in kidney epithelial cells, interacting directly with Sec10 [36], [37]. Cdc42 also functions in regulating apical-basal cell polarity by interacting with Par6, and this interaction in turn recruits and localizes aPKC to the membrane [38]. Since mRNAs encoding both Cdc42 and Par6 have CPEs in their 3′ UTRs, their localization and translation could be controlled by *orb2*. While we have not tested the effects of reducing *cdc42* activity, we found that a subset of the spermatid cysts are polarized in the incorrect direction in *par6* heterozygous males (data not shown).

The idea that *orb2* has other mRNA targets in cyst polarization would also be consistent with the effects of *orb2* mutations on asymmetric cell division in the embryo. We previously found that the accumulation of aPKC along the apical cortex of dividing neuroblasts depends upon *orb2* [30]. However, one of the other *orb2* asymmetric cell division phenotypes is a failure to properly orient the mitotic spindle. Since spindle orientation in dividing neuroblasts is thought to be independent of *apkc* [17], it would appear that the localized expression of other polarity proteins must also depend upon *orb2*. In addition to *cdc42*, another potential *orb2* target that has functions in spindle orientation would be the mRNA encoding Inscuteable (*insc*). In fact, Hughes *et. al.* [39] have shown that apical localization of the *insc* mRNA is important for Insc function in neuroblast cell division, and like *apkc-RA* mRNA it has CPE sequences in its 3′ UTR. Thus, a plausible inference from their studies is that Orb2 may help localize *insc* mRNA to the apical side of the neuroblast, and then activate its on site translation. (It is interesting to note that there are hints that mRNA localization may also be important when polarization is being initiated in epithelial cells [40]).

While *orb2* might regulate many of the same target mRNAs during both spermatid cyst polarization and asymmetric cell division, one apparent difference is in the penetrance of the phenotypes. Cyst polarization seems to be completely disrupted in the absence of *orb2* function. In contrast, only a subset of the cells in the embryonic neuronal and muscle cell lineages show obvious defects in asymmetric cell division. One likely reason for this difference is that polarization during cell division can be mediated independently by cross-regulatory interactions between factors specifying the apical and basal domains. Since several of the polarity proteins do not seem to be expressed in the spermatid cysts, it is possible that these cross-regulatory interactions either do not exist, or are not sufficient for polarization. Conversely, the fact that there are even modest cell division phenotypes in neuronal and muscle precursor cell lineages in *orb2* mutants also implies that these cross-regulatory interactions are not in themselves sufficiently robust to ensure that these cells always polarize correctly in the absence of *orb2* function.

In this context, it is worth noting that the role of *orb2* in both asymmetric cell division and spermatid cyst polarization seems to differ from many of the previously documented functions of mRNA localization in processes that depend upon polarization such as cell fate or polarity axis determination. In most of the instances in which mRNA localization is known to have an important role in cell fate or polarity axis determination (e.g., *prospero* mRNA localization to the basal daughter cell during neuroblast cell division or *oskar* mRNA localization in the specification of the posterior pole of the oocyte/embryo), the underlying polarity of the cell, cyst, egg, embryo or organ is pre-defined by the activity of an upstream and seemingly distinct polarizing pathway, most typically involving the aPKC/PAR machinery. The mRNAs encoding the cell fate or axis determinants function primarily in the elaboration or execution of this polarity decision, and not in the initial definition of polarity [41]–[43]. In contrast, *orb2* seems to be intimately involved in the upstream polarity pathway, helping the aPKC/PAR machinery define and then maintain the underlying polarity. In fact, in the process of correctly orienting spermatid cyst polarization the functional interdependence of *orb2* and *apkc* would seem to be mechanistically equivalent to the cross-regulatory interactions that underpin “classical” polarization by the aPKC/PAR machinery.

Other unresolved questions include the identity of the signals that initiate and orient cyst polarization relative to the testis itself. The latter, cyst orientation, most probably depends upon an external signal and the most likely source would be the two somatic support cells, the head and tail cyst cells, which encase the spermatid cyst (Fig. 1A). These two cells arise from a population of somatic stem cells at the apical tip of the testis and associate with the newly formed germline daughter cell that ultimately gives rise to the 64-cell spermatid cyst. The two somatic cells grow without division, surrounding the germ cells as they undergo mitosis and meiosis. Interestingly, one of the cells ends up on the apical (relative to the testes) side of the newly formed cyst and it expresses the Dlg guanylate kinase. The other is on the basal side and doesn't express Dlg [44]. We confirmed this observation and found that another polarity regulator, Bazooka, seems to exhibit the same expression pattern. Either one of these cells could potentially produced a signal(s) that orients cyst polarization. It is also possible that polarization depends upon an autonomous signal generated in the germline when the cysts commence differentiation. Further studies will be required to answer these and other questions.

## Materials and Methods

### Fly strains

*apkc* mutant alleles *apkc^k06403^, apkc^ex55^*, *apkc^ex48^* and *par1-GFP* are kind gifts from Dr. Elizabeth Gavis, Dr. Yu-Chiun Wang and Dr. Eric Wieschaus at Princeton University. *apkc* RNAi lines are obtained from Bloomington Drosophila Stock Center (stock # 34332 and 35140), targeting two different exons common to all *apkc* mRNAs (sequence: CTGGAGAAGACGATTCGTATA and CAAGCTGTTGGTGCACAAGAA) [45]. MTD multiple driver line is obtained from Dr. Andrew Hudson and Dr. Lynn Cooley from Yale University. The *orb2* mutant allele *orb2^36^* was generated in the lab [29]. *orb2^ΔQ^* is a gift from Dr. Barry Dickson from IMBA, Austria.

### Quantifying spermatid cyst polarity defects

Testes were dissected and fixed/stained following standard whole mount staining procedures [29]. DNA was dyed with Hoechst. Testes were further examined under epi-fluorescence microscopes. Percentage of testes with spermatid nuclei near the stem cell/spermatogonial region was recorded. Those were considered defective in orienting the polarization of the spermatid cysts.

### Immunocytochemistry/antibody staining

Whole mount staining was performed as in [29]. Antibodies used were as follows: mouse anti-Orb2 2D11 and 4G8 IgG (undiluted, developed in the lab) [29], rabbit anti-Bol (1∶1000, a gift from Steven Wasserman) [46], monoclonal anti-β-Tubulin E7 1∶50 (Developmental Studies Hybridoma Bank), rabbit anti-aPKCζ (1∶1000, clone c-20, sc-216, Santa Cruz Biotechnology), rabbit anti-GFP (1∶1000, Cristea Lab, Princeton University). Rabbit polyclonal anti-Dlg-PDZ1 (1∶1000) and guinea pig anti-Bazooka (1∶500) were provided by Yu-Chiun Wang [47]. Actin was stained with Alexa488-phalloidin (Invitrogen, Carlsbad, CA). DNA was stained with Hoechst (1∶1000). Secondary antibodies used were goat anti-mouse IgG Alexa 488, 546 or 647, goat anti-rabbit Alexa 488, 546 or 647 (Molecular Probes, Inc.). Samples were mounted in Aqua-polymount on slides for an inverted Zeiss LSM510 or Leica SP5 confocal microscope.

### Fluorescence *in situ* hybridization

Fluorescence *in situ* hybridization was performed as described in [29]. Fluorescent antisense probes for *apkc* and *orb2* were synthesized by Biosearch Technologies (www.biosearchtech.com) or synthesized in Tyagi lab. Forty non-overlapping 17 bp probes targeted at *orb2* mRNA sequence from cctggacgatcagatgt to atatgttatttaatctcac were synthesized and labeled with Quasar 670 and used at 1∶100 dilution. For combined *in situ* hybridization-antibody staining experiment, the *in situ* hybridization was performed first, followed by a sample re-fix and then standard whole mount antibody staining.

## Supporting Information

Figure S1 Baz, Dlg and Par1-GFP expression during spermatogenesis. Whole mount staining of wild type or *par1*-GFP testes with monoclonal antibodies against Baz (A, B), Dlg (C, D) or GFP (E, F). While Baz, Dlg and Par1-GFP proteins are observed in somatic cyst cells that encase the developing germline cysts (A, C, E, yellow box outlines one early spermatocyte cyst), there appears to be only limited expression in the germline cells, especially at stages when aPKC is highly expressed. Arrows in B, D and F indicate the tips of the elongating spermatid cysts where aPKC protein normally accumulate. Note that Baz. Dlg and Par1-GFP are not detected in this region of the cyst. All images are orientated with apical side of the testis to the left and basal to the right.
(TIF)

Figure S2 *apkc-RA* expression during spermatid elongation. A) *apkc-RA* is not detected in the mitotic or meiotic spermatocyte stages (SMC). It first spears at low levels in the spermatids that just completed meiosis (early SPT), while its levels gradually increase once the spermatids begin to differentiate (late SPT). In the merged panel on the far right: green, *apkc-RA* mRNA; red, Orb2. B) At early stages of spermatid elongation as the cyst polarizes, *apkc*-*RA* accumulates at higher levels at the apical (with respect to the testis) side of the cyst (arrow) while the spermatid nuclei cluster at the other, basal side of the cyst (arrowhead). Insert in B is a zoom-in view of the boxed region. In the merged panel: green, *apkc*-RA; blue, DNA. Scale bar: 50 λm.
(TIF)

Figure S3 Orb2 binds to sequences in the *apkc-RA* 3′ UTR *in vitro*. Lanes in left half: Recombinant Orb2-RRM (Text S1, Supplemental Methods) was added as indicated (0.05 µl–1.0 µl) to the reaction mix containing the either the *com*-GS 3′ UTR probe (Probe C) or the CPE containing *apkc-RA* 3′ UTR probe (Probe A). The C probe is derived from a common 3′ UTR (see Fig. 3 and Text S1, S2) and doesn't contain a CPE sequence. The *apkc*-RA probe A is derived from the unique RA 3′ UTR and contains the first CPE site (see Fig. 3 and Text S1, S2). Lanes in right half: Increasing amounts of cold competitor as indicated were added to the incubation mix (left to right: -, none; 100 fold excess; 200 fold excess; 400 fold excess). Competitor Comp-CPE is derived from the RA-3′ UTR and spans one of the 3 CPE sequences. Competitor Comp-com is derived from the common 3′ UTR sequence (see Fig. 3A and Text S1, S2).
(TIF)

Text S1 Supplemental materials and methods. EMSA assays and primers for generating probes. Immunoprecipitation and RT-PCR procedures and primers for detecting specific mRNAs.
(PDF)

Text S2 Sequences of the *apkc* and *orb2* mRNA 3′UTRs, and of the probes/competitors used in EMSAs.
(PDF)
