# Supplementary figures and images for: Spermatid Cyst Polarization in Drosophila Depends upon apkc and the CPEB Family Translational Regulator orb2
Source: PLoS Genet. 2014 May 15;10(5):e1004380. doi: 10.1371/journal.pgen.1004380 (PMC4022466; doi:10.1371/journal.pgen.1004380)

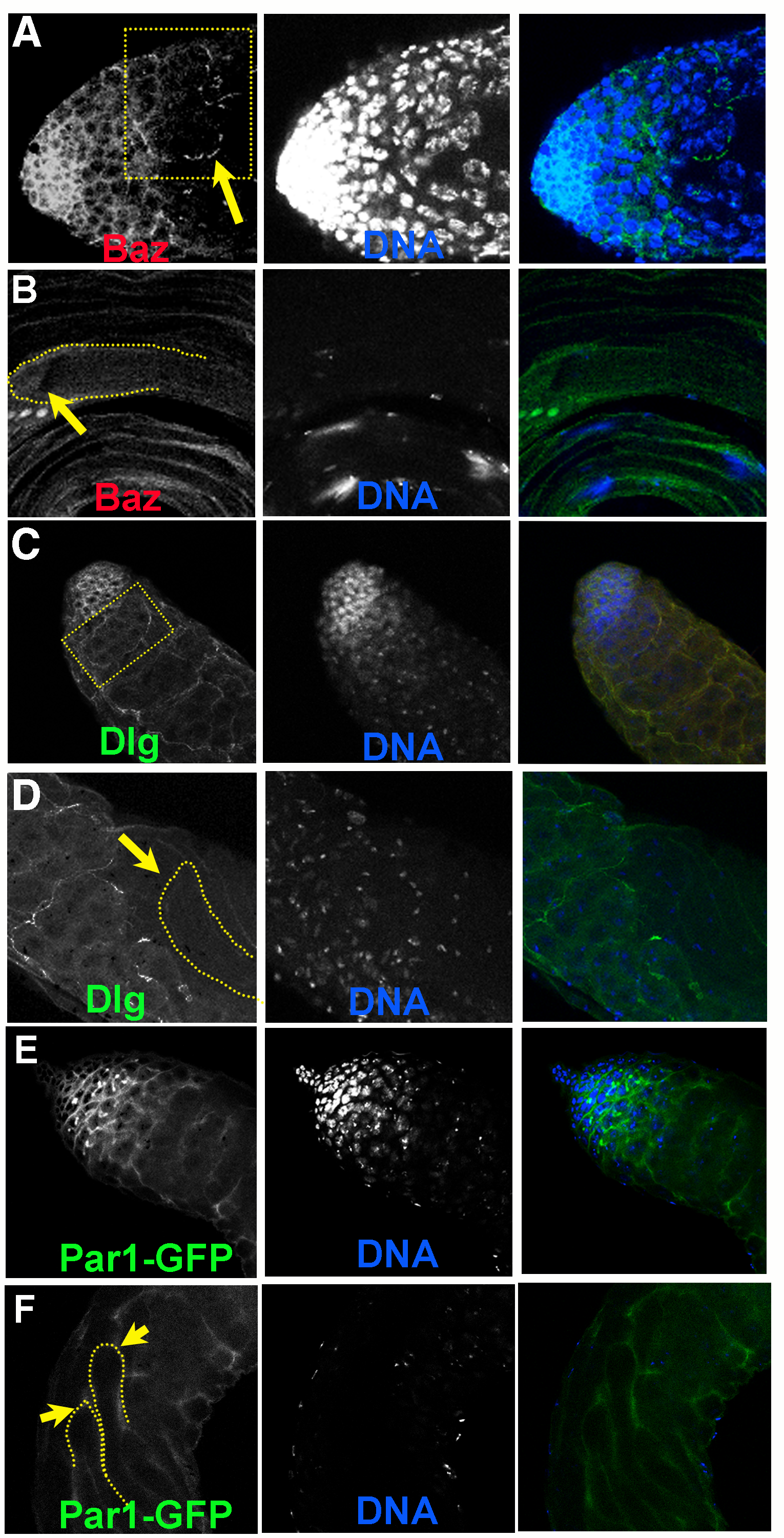

Supplement: Figure S1 — Baz, Dlg and Par1-GFP expression during spermatogenesis. Whole mount staining of wild type or par1-GFP testes with monoclonal antibodies against Baz (A, B), Dlg (C, D) or GFP (E, F). While Baz, Dlg and Par1-GFP proteins are observed in somatic cyst cells that encase the developing germline cysts (A, C, E, yellow box outlines one early spermatocyte cyst), there appears to be only limited expression in the germline cells, especially at stages when aPKC is highly expressed. Arrows in B, D and F indicate the tips of the elongating spermatid cysts where aPKC protein normally accumulate. Note that Baz. Dlg and Par1-GFP are not detected in this region of the cyst. All images are orientated with apical side of the testis to the left and basal to the right. (TIF) [file pgen.1004380.s001.tif]

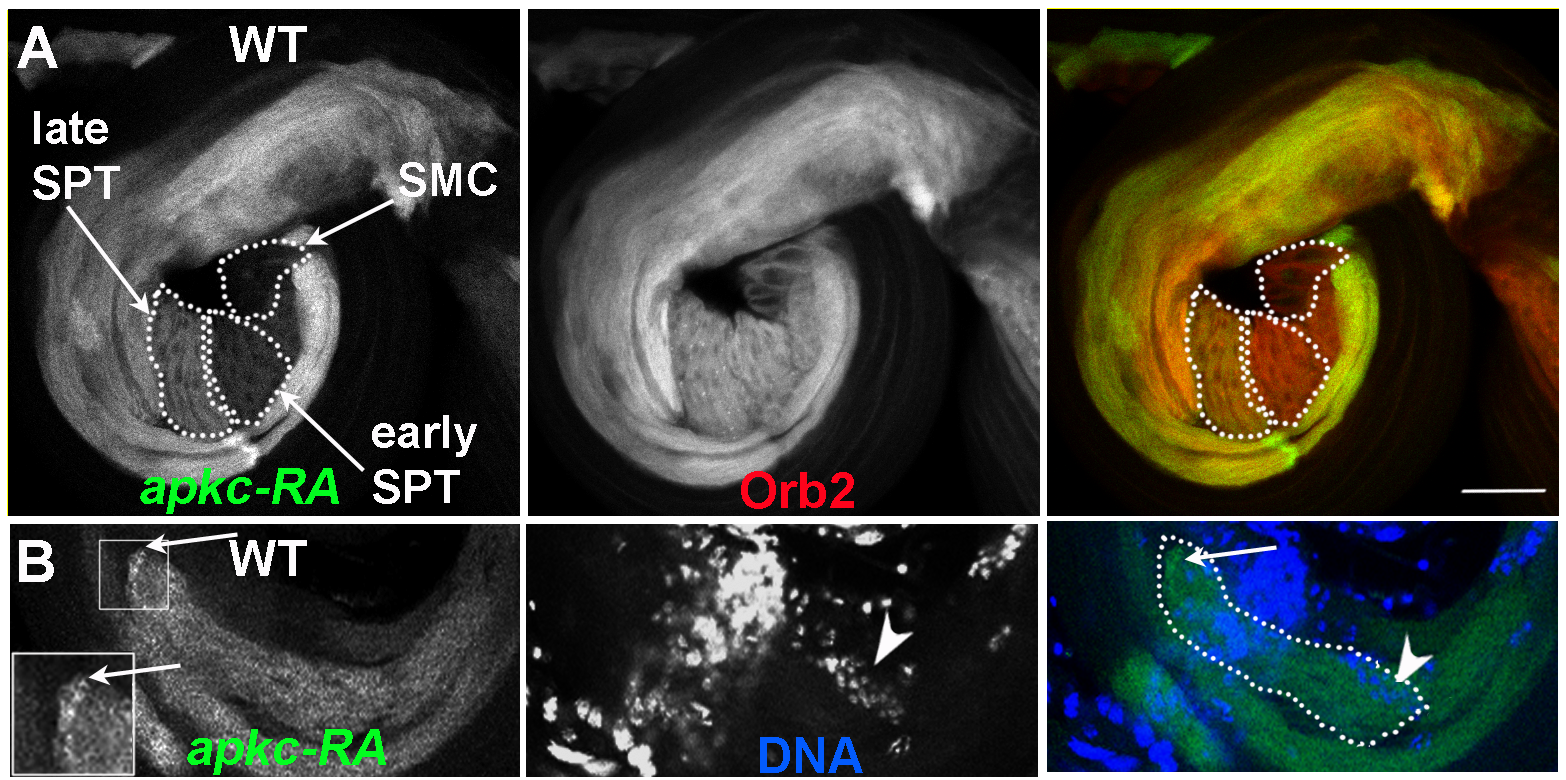

Supplement: Figure S2 — apkc-RA expression during spermatid elongation. A) apkc-RA is not detected in the mitotic or meiotic spermatocyte stages (SMC). It first spears at low levels in the spermatids that just completed meiosis (early SPT), while its levels gradually increase once the spermatids begin to differentiate (late SPT). In the merged panel on the far right: green, apkc-RA mRNA; red, Orb2. B) At early stages of spermatid elongation as the cyst polarizes, apkc-RA accumulates at higher levels at the apical (with respect to the testis) side of the cyst (arrow) while the spermatid nuclei cluster at the other, basal side of the cyst (arrowhead). Insert in B is a zoom-in view of the boxed region. In the merged panel: green, apkc-RA; blue, DNA. Scale bar: 50 λm. (TIF) [file pgen.1004380.s002.tif]

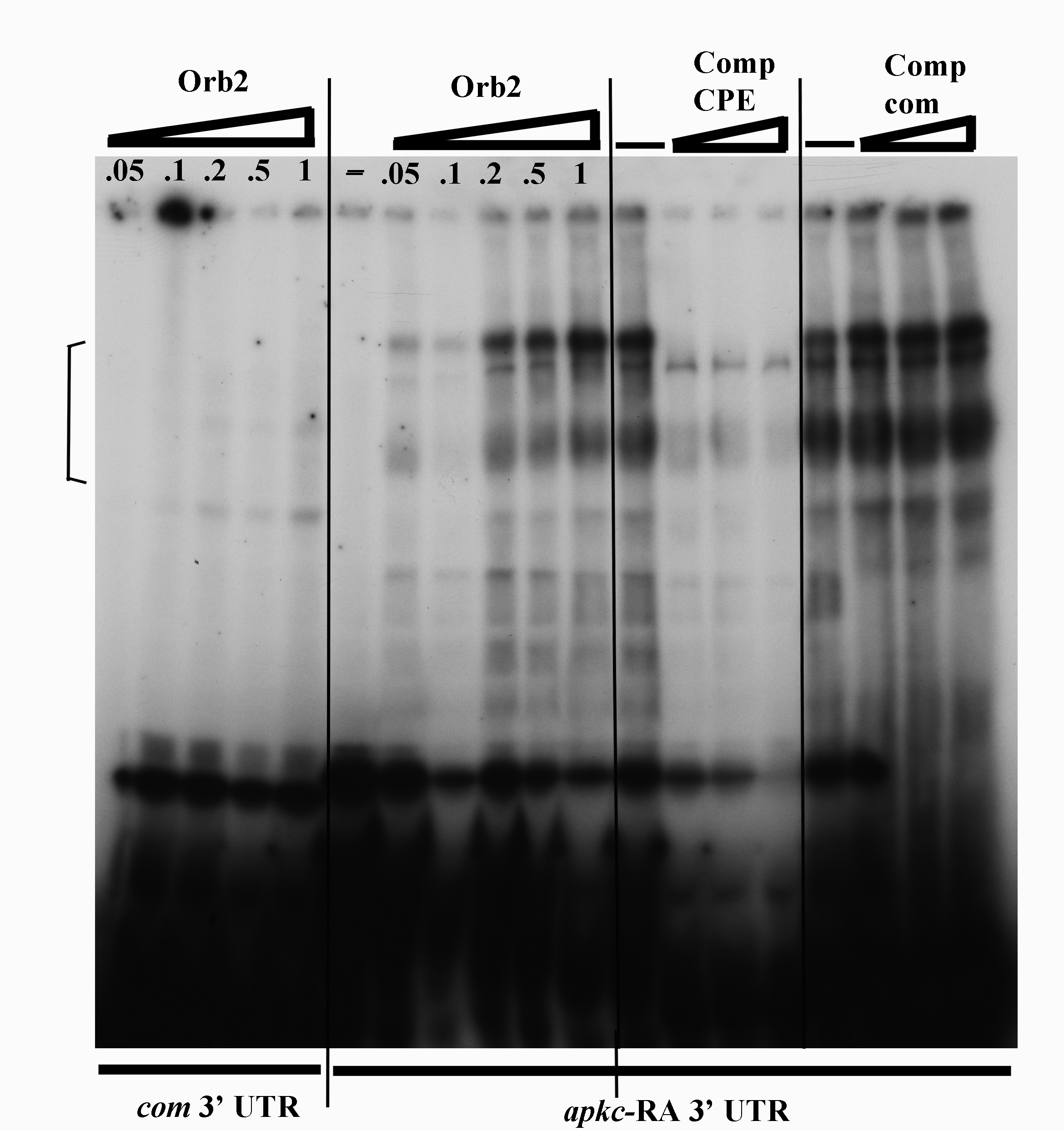

Supplement: Figure S3 — Orb2 binds to sequences in the apkc-RA 3′ UTR in vitro. Lanes in left half: Recombinant Orb2-RRM (Text S1, Supplemental Methods) was added as indicated (0.05 µl–1.0 µl) to the reaction mix containing the either the com-GS 3′ UTR probe (Probe C) or the CPE containing apkc-RA 3′ UTR probe (Probe A). The C probe is derived from a common 3′ UTR (see Fig. 3 and Text S1, S2) and doesn't contain a CPE sequence. The apkc-RA probe A is derived from the unique RA 3′ UTR and contains the first CPE site (see Fig. 3 and Text S1, S2). Lanes in right half: Increasing amounts of cold competitor as indicated were added to the incubation mix (left to right: -, none; 100 fold excess; 200 fold excess; 400 fold excess). Competitor Comp-CPE is derived from the RA-3′ UTR and spans one of the 3 CPE sequences. Competitor Comp-com is derived from the common 3′ UTR sequence (see Fig. 3A and Text S1, S2). (TIF) [file pgen.1004380.s003.tif]
