# Supplementary material for: Spermatid Cyst Polarization in Drosophila Depends upon apkc and the CPEB Family Translational Regulator orb2
Source: PLoS Genet. 2014 May 15;10(5):e1004380. doi: 10.1371/journal.pgen.1004380 (PMC4022466; doi:10.1371/journal.pgen.1004380)
Supplement: Text S1 — Supplemental materials and methods. EMSA assays and primers for generating probes. Immunoprecipitation and RT-PCR procedures and primers for detecting specific mRNAs. (PDF) [file pgen.1004380.s004.pdf]

MATERIALS AND METHODS:

Electrophoretic mobility shift assays (EMSAs): EMSAs were performed essentially as described in [1] except the followings: Orb2 C-terminus including the two conserved mRNA binding domains and the zinc finger domain was fused to GST. The fusion protein was expressed in *E. coli* and purified with glutathione beads. GST expressed and purified under the same conditions was used a negative control, which did not bind to any of the probes (not shown). Purified proteins were tested for purity and concentration with SDS-PAGE gel and Coomassie blue staining, and adjusted to 2λg/λl stock.

apkc-RA sequence containing three CPE elements (from *apkc*-RA specific 3' UTR) and *apkc-com* sequence without CPE (from a common UTR sequence to all *apkc* mRNAs) were PCR synthesized from wild type fly RNA extract reverse transcribed with an oligo-dT primer. PCR products were ligated into pCR® II vectors and sequenced to confirm amplification fidelity. A subfragment (of ~150bp) from each recombinant was PCR amplified from the plasmid with forward probes containing the T7 promoter sequence and reverse probes (PCR primer sequences are shown below). Products were gel purified and sequenced. ~400ng of product was used as a template for *in vitro* transcription using T7 RNA polymerase and [α-32P] UTP. Products were purified with GE-mini-spin column. 1/40 product probe was used for each binding reaction before series dilution. Binding reaction happened in the following buffer: 25mM Tris (ph7.5), 10mM EDTA, 0.1mM DTT, 2 µl RNase inhibitor (NEB), 4% Ficoll and BPB, 0.25mg/ml BSA, 100mM NaCl, 0.5µg/ml tRNA. Mixture was incubated for 30 min at 22° C before loading onto a 4% acrylamide (mono/bis, 29:1)-0.5× TBE-2.5% glycerol gel. Electrophoresis was done

under 4°C and 180 V for 3 to 4 h using 0.5× TBE-2.5% glycerol as a running buffer. Gels were dried and exposed to BioMax MR film (Kodak) for 20 hours to 5 days.

Primers used:

For amplifying whole *apkc-RA* 3'UTR or *apkc-com* 3'UTR:

apkc-RA 3'UTR: CAAGCAAACACAATCCGTCTACTG and

GTCTTATACAATACTTTATTCGTTTTTCT

apkc-com: ACCACGAAATGTGCGACTTACATCC and

GCTTGCATTTGATTATTATGAATGTTATTG.

For synthesizing gel shift probes:

apkc-RA probe (final probe 155bp): TAA TAC GAC TCA CTA TAG GGA GAC ACA ATC CGT CTA CTG GCA

and GAT GCT GGC ACT ATA TAT GCC ATC AC.

apkc-com probe (final probe 145bp): TAA TAC GAC TCA CTA TAG GGA GAC TTA CAT CCG TAT AAC ATG C and GCT GCT GGC TGA GAT TGT TAT TGC T

Immunoprecipitation and RT-PCR: Immunoprecipitation was performed essentially as described before [2], except the followings: monoclonal anti-Orb2 antibodies 2D11 and 4G8 supernatants were affinity purified with Orb2 coupled HiTrap NHS-activated HP column (GE healthcare) before used for immunoprecipitation. Purified Orb2 antibodies were mixed with testis extract. After 0.5h-2h incubation at room temperature, protein-A/G-agarose beads (Calbiochem/Millipore) were added in; the mixture was then incubated at 4C° for 2h to overnight. After four washing, mRNA was extracted from the

beads with phenol-chloroform followed by ethanol precipitation. RT-PCR was done according to [2]. Reverse transcription for detecting *apkc* was done with an oligo-dT primer (IDT); for detecting *orb2* was done with an *orb2* specific 3'UTR primer. Primers used were as follows:

Primers used in RT-PCR experiments:

oligo-dT: TTTTTTTTTTTTTTT (IDT ReadyMade^{TM})

apkc PCR primers:

PF: CAT CAA CAT CAG CAG CAA CAG CAG
PR: GTT GTT GTT GTA AGT ACA GTG GAA TGC A
1: CTT GAA TGG TGG CGT GAC CTG TTT
2: ATA GCT CAA AAG GAG GTG CAA CCG
3: CGG TTG CAC CTC CTT TTG AGC TAT
4: ATC GGG AGT CCG CGT TCA TGG ATA T
5: GGC GCT TGA GGA ACA TTG GGA AAT
6: TGC TGA CAT GTT AAC AGG CCT CTC G
7: ATG ATC ATC TGG AGT GGC TTC TGC
8: CTC CGC GGC ACA TGA TGT CCG ACA
9: TTC CAA TAA GAA TTG CCC AGC GGC
10: ATG GAA TTG GAC GAG GCC ATA CGA

orb2 primers:

reverse transcription: AGA GCC TCG ATG GCG AAC CCA
PCR: TTG TGT GTG ATT GTG AGT GTC CGT and
GCG CAT CTC CGC CAC CAG TT

Reference:

1. Aoki T, Schweinsberg S, Manasson J, Schedl P (2008) A stage-specific factor confers Fab-7 boundary activity during early embryogenesis in Drosophila. Mol Cell Biol 28: 1047-1060.
2. Xu S, Hafer N, Agunwamba B, Schedl P (2012) The CPEB Protein Orb2 Has Multiple Functions during Spermatogenesis in Drosophila melanogaster. PLoS Genet 8: e1003079.
