# Supplementary material for: Spermatid Cyst Polarization in Drosophila Depends upon apkc and the CPEB Family Translational Regulator orb2
Source: PLoS Genet. 2014 May 15;10(5):e1004380. doi: 10.1371/journal.pgen.1004380 (PMC4022466; doi:10.1371/journal.pgen.1004380)
Supplement: Text S2 — Sequences of the apkc and orb2 mRNA 3′UTRs, and of the probes/competitors used in EMSAs. (PDF) [file pgen.1004380.s005.pdf]

***apkc*-RA 3' UTR with three CPE sequences in bold and underlined:**
CAAGCAAACACAATCCGTCTACTGGCAATATAACTTTGTTAAGTATTAAAAA
AAAACTTTTGT**TTTTAAT**GCATTCCACTGTACTTACAACAACAACAAAAACA
TATATATATTTATTATTTTTGTTTATTTGTGATGGCATATATAGTGCCAGCATC
TTTTTGTAATCCACATATGAATATTACGAAAAAATTATTAAAATTAGTATATA
TCAAAAATATCATTGTGTTGCGAAGGTAAATAAAATATTGTGT**TTTTAAT**TG
AAGCTAAAGATAAATTTCAGCAAAAATGTTACTTGTTAATGTATGAAAAGCA
AATAAAAGAGAACAAATTAAGCGCATATTGTTAAATTTCCAAATACAACGCA
ATCTAAGAAAGTAATATTGATCAAAGTTTTTGATTAAAGAACATAGCAAGCA
ATCAATTTAAATGCAAAACAAACAGGACAAACTGAAAAACAATTTCAAATTG
CACTTAAACGTTACCGA**TTTTAT**TAGTAACAACCAATATTCTTTAGCCTATCC
CAATTATTATGTTGTTGTGTTTGATAACCAAGTTCTATGAGTAAGTTTAATTA
GTATATAATGATTTTACTGAGCGTATAACATCAATTTGTATCTTCTTCAACTG
ATGTTTCCGACTAATTTTTTAAACTGGATGCACTTTTGGCATATACTACCAGA
TGCTAGCAAAATGAAATTGAAAACGTAAACGATAGTTGCGTTACAATTATGC
ATATAAAGAAAAACGAATAAAGTATTGTATAAGAC

***apkc* Gel shift probe sequences:**
apkc-RA probe:
GCAAACACAATCCGTCTACTGGCAATATAACTTTGTTAAGTATTAAAAAAAA
ACTTTTGT**TTTTAAT**GCATTCCACTGTACTTACAACAACAACAAAAACATATA
TATATTTATTATTTTTGTTTATTTGTGATGGCATATATAGTGCCAGCATC
apkc-com probe:
ACCACGAAATGTGCGACTTACATCCGTATAACATGCGGCTCTATGGCGAGGA
TTCCAGTGATTACGATTCCGTGGCTGACGATTTGAGTCTCCTGCATTTGAACA
GCGCAGCGGGAGCTGGTGGATCAGTTAGCAGCAATAACAATCTCAGCCAGCA
GC

Gel-shift competitor sequences:
CPE containing competitor (competitor A):
ACU UUU GUU UUU AAU GCA UUC CAC
Non-CPE containing competitor (competitor N):
GUG CGA CUU ACA UCC GUA UAA CAU G

***orb2*-RH 3'UTR with thirteen CPE sequences in bold and underlined** (other *orb2* mRNA isoforms have shorter 3'UTR that are part of the RH 3'UTR sequence):
CGGCGGCGCTGGTAGGCCGCGCTGCACGACGAGAGACGCCAACAGAGGGAG
CAACGACACCGGTTGTTAAACGCAGCGCTGGCAGTGCCCAGAATCATAACAG
AATCATCAGGATCGGGAGGAGTCGCAGAAGGAGCGGGAGTGGCAGCACTAG
ATGCCGCTGGCGGAGATGCGGATCGAAGGCGAAGAATCTAGTAGATAACCAT
TAGAGACAACACACGCACCACCAACACCCACTCACACACAACAGTCGGATAC
TATTCTCATAAGCAAAAGACAATTGCCCGGGGGAATCACCCATTCCGAAGGG
CAAGGTGCAAATACAACGGGAGCTGGACAAAGGTGTCCCTTTGAATAGCCAC
CAGAGTCTACAGTTTTAGAGGCGAGAAACGAAAGCAACATAGTTTTTAGTTC
TTATTTATTCGGCAATCAGTTTTTTTTTTTTGTTTCGCGCTTGCTAGTTTTACAT
TTGCTTAACCGAAATTTTAGTTTTGATTTTGCGTTCGATGCCTGCACAGTATTT

TTTGTACTTTATATTTTTAGCAACATTTATGTTTGCTAATATGTTATTTAATCT
CACAAAAGAAAAAAAAAAAGGAACCCTCGTAGTCAGCCAATTTCCTCGCAAA
GGGCCGTTTACATATATTATAATCTTTTTGTAACTAGATAACTTAGTAGCCAT
CATCTTGTATGCCAATCGAGGCTAGTCCTAAAATTATTAGTGAGCTGCTCACC
CTCTCTCGAAAAGGCTTTTTGCTTGAGTGTGCAGGGGCTGTCCTTAAGTTCCT
AAATTGTGACAAAATCAAAACTACATCGGTTTTTGTATTAGCCCTTTCCGAAA
TTAGATCAATTTATAAAATTCTTACAAAAGACTTTAGAAAAGACAATATGCA
AAATGTCTGACATTTGTATGCTAGCCATTTCTTACGACACAATTGGTAAGAGA
TCGCTTAATCATATCATATCTGCATACAGTTATAATCCCTATTTATTTTTGTTT
AATCCTTACATAATACGGTCTATTAGCATTCCCACTGAAGAATCGTATATATT
ATCTGTGCAATATATTCGATTGCTTCTCCGTTACATGCTGCTAATTCTTGCTTA
TCTAACACTTATATAATTATTGTATGTGAAAATCGTACAAAGCTTATAAAACA
CACAAAACCAATAGGTGGCGATTGCTTCCCCGTTACATGCTACTTATTCCTGC
TTATCTAATTATATAATTAGGGTATGTGAAAATCGTACAAAGCTTATACACTT
ACTAAACGATTCTTAACTTGTTACCACTCCTGCCAATAGGTGGCTTTCACTAC
GATTTTCACACGACTCCG**TTTTAT**GTTTTGCTGGCACATTGAAATCACACTAA
TTAATGAGTTTAAACCCCACACTCTGAGATAGGTTCTTCTTTCCTAATATTGA
TGGCTCAT**TTTTAAT**TATAATCTAGTCCACACTCATACATACACGTACGTACA
CAGACAACAACAACAACACATTTACACTGAAAGTCTGTTTGCATTTGTTGCCA
TTTGGCTTAATGAAGAAAAAATAATAATAAAAATATACATATAATTAGAACT
AAAATGTAGAAAAAAACAAAACAGTCAAATTGCTTGTGTTCAGTATTATGAT
TTATTTATGATTTTCCACTGAAATGTCATATTTTGTTTATTTATTAACCAATTG
TTTTTGTTATTTAATTTACGGCGTGTTGCTAACTAAGGGACAGCGGGCTCACC
TCCCGAAGCTATAGAAGAACGTATATATTATTGCAAATATATAAATATATCTG
TAATGTAAAAAGTAAT**TTTTAT**ATACGACATACATCTATTAACACTATGAAT
ACTTGTATTAGACAACT**TTTTAT**ATAGAAACTTTTGTATAAACTTTT**TTTTAT**
TACTCTTATGATTATTCAGAAGTTAGTTTAGTCCAAACAACTGAATACAAAAA
TAAAACCAAAAACGCGAAGATAAATAGACATGCTAAAACATTTAAAGAAAA
TTTAAATATATACAAATTATGTGCAATACTATTTCGTGAACGATTCAAGCGTA
TTATTGAATACCAAATATATAAACAACTTAAAAAAGATATGTATCTGTACAA
ACATAACGTATACAAGATAATTGCAATCGTATAACCAAAGTAGTCAAATATT
ACCAAAACCCAATGGTTCCCCAAACTTTCAACTAATTGGCATGCGTTTTTTGA
CATGCGAACAGGGCTTAAAGTTCGCGTTTGGTTATTTCCCCGTGAATCCTAAC
TATAAAAATCAAATGGAATATCTATCTATACCTAGGGCAATACTTATGGAAA
ATTATCAAAATGTAAATCGAGCAAAATTATTTTGTAAATTATACGCGTTAAGG
AACGAGACAAAATTTGCTACGGTGCATGCAAACAATACGATATTTTTTCTATA
CTACCAGCAAAAGTACTAAAGATCTAATTCGAATTGATAAACCAAAAACGTG
AGAGGTCAAGTCGCTTTGGACTTCTATGATATAATTAATAAATAATAGTCAA
ATCTATTAGCAAATTTTTGCTCGCGCTTTCTTTACAAGACAACAAAAAAAAAA
AAAAGAAAAAACAAAATTTAAGTCATGCAAAGTTGTAACTCAAGTTGATTGC
GTGGCACTTTCAAAGAAAAACAAGTTTATAGCAATTTTTCTAGAAT**TTTTAT**A
TTACAATGAACTATTGAATATGTATCATATATATATACTACATATATATTTTTT
AAACCGCAAAACCAATTAATTAGTGGACTCGAAGCGTTCGTTTGTTTTGGTTA
TAATTTATTACTTTTAAATACTTACCGAAATATCGTGTATTAAAAGCGAATGA
TTCTACACAAAACAAAAACCAACAAGCCCAATTAAATATTATATCAAATACA
ACAGCCTGCTCTATGTAGTGTTAAACAATTTGTGCAAATATGTTCTAAAACTT

TCAAAGTAATTAAATGCTAGTCTTTATAAAGGCAAAATCTGTTACAAAAGCA
AACACGGAACGGAAACGGAAATCGGTTAGCGGTAAACGGCAATCGGTCAAT
GGCTATCGATAACTGTGCGTAATGATGGTGTTTTTTTAAATGTAGTAAAATTT
AATA**TTTTAT**AGAT**TTTTAT**AC**TTTTATA**TAACATATACTTTATATTTTACCT
GTTTAACAAAAACTCAATGAAAATGTTAAAAAAAACTTAATGTGCTATAAAG
AAGTAACTTATCAAACAAATATTTTACTATACCAAAAAAAGTTGATAGAGAT
GAGAATTCTTTACGGAAAACCGCTCAATGTTTTTGCTGCTAACGACGTAGGA
ATCTGGAATCTTGTGTAGCGTGTTAAAAAAATATTTATATCAATAAGCAATCC
TTAATCCTTTTTGTTCTGTTTCATTATTTATTTTGTTTCCCGAGTATTCCATAAA
AATTAGGCAAAGACGTTTAAGTTATCCTACTTCTGAGCTTGGATTACGGATAT
AAGTCGGATTGACAGCTTTAG**TTTTATA**GAGAATCTTGTTATAGATAATGTTT
ATGTTGACTT**TTTTAT**ATGGATCCTGTTATTATACCGATAATGACAATGAAAA
TTTGTATGCCTAATTCTTAAATATATTTTTGTATCTTTTGTAGTTGCAAGTAGT
TTCCTGGTAGTTTAAAAAAAAAAAACAAACTCCGTTGAGACAT**TTTTAT**ACTTT
GTTTATTC**TTTTAT**ACGTATACATATTTTTAAAAAAATATGACAAAAAACAAT
ATTTGATATATACACTTTATACAAAAGAAAAAATGGAAAATGAATGAAAAAA
AGTTTTTGAAATTAATAAACGGACATAAAGT
